# Discovering high-efficiency cathode presodiation additives for Na-ion batteries via high-throughput screening

**DOI:** 10.1126/sciadv.aed4045

**Published:** 2026-05-15

**Authors:** Di Wu, Yan Wu, Huiling Huang, Minfei Fei, Jingyang Wang, Qian Qiu, Hao Chen, Wen Xu, Xiaoyan Lin, Tong Shen, Zhengyan Lun, Yan Jin, Jia Zhu

**Affiliations:** ^1^ School of Sustainable Energy and Resources, National Laboratory of Solid State Microstructures, College of Engineering and Applied Sciences, Frontiers Science Center for Critical Earth Material Cycling, Collaborative Innovation Center of Advanced Microstructures, Nanjing University, Suzhou 215163, Jiangsu, P. R. China.; ^2^ School of Materials Science and Intelligent Engineering, Nanjing University, Suzhou 215163, Jiangsu, P. R. China.; ^3^ Suzhou Laboratory, Suzhou 215123, P. R. China.; ^4^ School of Chemical Sciences, University of Chinese Academy of Sciences, Beijing 101408, P. R. China.; ^5^ Binzhou Institute of Technology Weiqiao-UCAS Science and Technology Park, Binzhou 256606, Shandong Province, P. R. China.

## Abstract

Presodiation represents a crucial strategy for compensating the capacity loss and boosting energy density in practical Na-ion batteries. Through high-throughput screening, we identify 52 promising candidates as potential presodiation additives. Experimental validation confirms that several screened compounds, including Na_4_FeO_4_ (NFO), Na_4_TiO_4_, Na_5_FeO_4_, and Na_5_NiO_4_, exhibit high compensating capacities up to 537 milliampere-hours per gram (mAh g^−1^). Notably, NFO delivers an irreversible capacity of 451 mAh g^−1^, and, critically, 94.5% of its capacity is delivered below 4 volts. The presodiation mechanism of NFO is comprehensively studied by multiscale investigations combining DFT, DEMS, and synchrotron XRD/XAS. Full-cell tests of NFO demonstrate its universal efficacy across diverse Na-ion cathode chemistry, exhibiting boosted energy density and cycle stability. In particular, the incorporation of NFO increases the initial discharge capacity of the O3-NFM full cell from 109.4 to 141.3 mAh g^−1^, with 83% retention after 200 cycles. This work not only establishes several highly effective cathode presodiation additives but also provides a variety of promising candidates for future research avenues.

## Teaser

High-throughput screening with experimental validation guides the rational development of presodiation materials.

## INTRODUCTION

Na-ion batteries (NIBs) as sustainable complements to Li-ion batteries (LIBs) hold substantial promise for large-scale energy storage applications due to not only the abundant reserves of sodium (*1*, *2*) but also the wide range of cobalt-free cathode chemistries (*3*, *4*). However, the commercialization of NIBs is hindered by their relatively low energy density. In full-cell configurations, the sodium reservoir is limited because active Na ions are provided exclusively by cathodes. On one hand, the mainstream Na-ion anode, hard carbon (HC), often shows low initial coulombic efficiency (ICE; 70 to 80%) (*5*, *6*) compared to that of graphite (~90%) (*7*), because of the formation of solid-electrolyte interface (SEI) and intrinsic defects (*8*, *9*). The low ICE results in a serious consumption of the limited Na^+^ in the first cycle and, therefore, substantially lowers the full-cell energy density. On the other hand, the SEI in NIBs exhibits relatively poor stability (*10*), making it prone to dissolution and reconstruction during subsequent cycles, further exacerbating the consumption of Na ions. Besides, many promising Na cathodes can only be synthesized in a Na-deficient state. For instances, P2-type layered oxides, featuring face-sharing Na sites and fewer phase transitions during Na deintercalation, are generally believed to offer superior rate capability and cycling stability (*11*). However, P2 materials (Na*_x_*MO_2_) can only be made with certain Na deficiency (*x* ≈ 2/3), substantially limiting their applications. In addition, many polyanion cathode materials like Na super ionic conductor also have Na vacancies in their as-synthesized states (*12*), making active Na loss a serious challenge for NIBs.

**Fig. 1.**
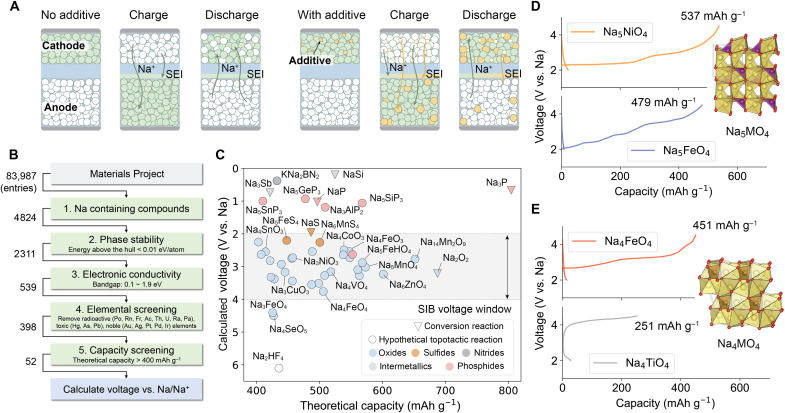
High-throughput screening for presodiation additives. (**A**) Schematic illustration of sodium compensation mechanism. (**B**) High-throughput screening workflow. (**C**) Distribution of the 52 compounds identified as potential sacrificial additives. SIB, sodium-ion battery. Crystal structures and 0.1 C first charge-discharge profile of Na_5_MO_4_ (**D**) and Na_4_MO_4_ (**E**) materials.

To compensate the irreversible sodium loss, presodiation is regarded as an effective strategy that has drawn growing attention recently (*13*–*15*). Presodiation refers to the introduction of additional sodium sources into the full cell, which can be achieved through either cathode or anode presodiation. Cathode presodiation using self-sacrificial additives has been widely studied because of its operational simplicity and compatibility with the current battery manufacturing process (*13*, *16*). As shown in Fig. 1A, self-sacrificial additives are often Na-rich compounds that supposed to decompose and release a large amount of active Na^+^ to compensate the Na loss during the initial charge. Ideal cathode sacrificial additives should exhibit (i) a high irreversible capacity far exceeding the capacity of cathode; (ii) a moderate decomposition potential within the cathode voltage window (typically 2 to 4 V versus Na metal); and (iii) stable decomposition products that do not trigger detrimental side reactions, ensuring long-term cycling stability. In addition, these additives should be made from earth abundant elements to align with the cost-effectiveness of NIBs.

Many sacrificial additives have been successfully used in LIBs such as Li_6_CoO_4_ (*17*) and Li_5_FeO_4_ (*18*), both of which have achieved remarkable efficacy in full cells. However, the current presodiation additives are rather less efficient: Although several Na sacrificial additives, such as NaCrO_2_ (*19*), Na_2_CO_3_ (*20*), Na_2_O_2_ (*21*), Na_2_NiO_2_ (*22*), and NaN_3_ (*23*), have been proposed, their practical application still faces various challenges. For instance, NaCrO_2_ has a relatively low theoretical specific capacity (around 250 mAh g^−1^); Na_2_CO_3_ has a high theoretical capacity of 505 mAh g^−1^ but suffers from poor utilization (<20%) at 4.5 V; Na_2_O_2_ delivers an actual capacity of 400 mAh g^−1^, yet its average decomposition potential is relatively high (>4.1 V); and NaN_3_ delivers 310 mAh g^−1^ capacity at 4.3 V, yet the safety concerns restrict its applications. Organic compounds as sacrificial additives were also investigated, such as Na_2_C_2_O_4_ (*24*), Na_2_C_4_O_4_ (*25*), EDTA-4Na (Tetrasodium ethylenediaminetetraacetate) (*26*), DTPA-5Na (Pentasodium diethylenetriaminepentaacetate) (*27*), and SM-2Na (Disodium malate) (*28*). While they offer reasonable capacities, their decomposition potentials remain relatively high. The oxidation potentials of EDTA-4Na, DTPA-5Na, and SM-2Na are all close to 4 V. Given these limitations, the development of alternative presodiation additives that simultaneously deliver high irreversible capacity at a low decomposition voltage constitutes an imperative priority for enabling the implementation of presodiation in practical NIBs.

Herein, we use high-throughput screening to comprehensively explore the chemical space of sodium-rich materials and successfully identify 52 promising candidates as presodiation additives. Several predicted materials, including Na_4_FeO_4_ (NFO), Na_4_TiO_4_, Na_5_FeO_4_, and Na_5_NiO_4_, were successfully synthesized, demonstrating high irreversible capacities with low average decomposition potentials. In particular, we further investigate the Na compensation mechanism of NFO using density functional theory (DFT), providing theoretical fingerprints of the decomposition products, which were also validated by systematic experimental characterization, including differential electrochemical mass spectrometry (DEMS), ex situ synchrotron x-ray diffraction (XRD), and ex situ x-ray absorption spectroscopy (XAS). We demonstrate that the incorporation of 15 wt % NFO additives elevates the initial discharge capacity of the HC||NaNi_0.33_Fe_0.33_Mn_0.33_O_2_ (NFM) full cells from 111.2 to 141.3 mAh g^−1^, leading to a 14.7 to 24.5% increase in energy density (with respect to the mass of both electrodes) while maintaining 83% capacity retention after 200 cycles.

## RESULTS

### High-throughput screening for potential presodiation additives

High-throughput material screening is first used to rapid identify potential presodiation additives, with the flowchart shown in Fig. 1B and the screening results in table S1. Starting from the Materials Project database (*29*), 4824 Na-containing compounds were initially selected. To ensure the synthesizability, we then applied a phase stability filter that excluded compounds with the energy above the convex hull (*E*_hull_) ≥ 0.01 eV. As *E*_hull_ is a measure of materials’ thermodynamic stability against decomposing into stable phases in the relevant phase diagram, we only considered thermodynamically stable materials (*E*_hull_ = 0) and materials with moderate metastability (0 < *E*_hull_ < 0.01 eV), yielding 2311 compounds. To ensure a low decomposition overpotential, the sacrificial additives should have reasonable electronic conductivity; therefore, we screened for materials with a theoretical bandgap between 0.1 and 1.9 eV, which returned 539 compounds. The upper threshold of 1.9 eV was chosen with reference to the polyanionic cathode Na_3_V_2_(PO_4_)_3_ (NVP), while the lower threshold was set to exclude metallic phases that are generally chemically incompatible with high-voltage cathodes (note that we also performed a screening with the lower threshold extended to 0 eV; these additional compounds are summarized in table S2). While a small bandgap almost assures that the materials are electronic conducting (*30*, *31*), compounds with large bandgap may also exhibit electronic transport when certain point defects are present (*32*), which may be considered in the future study. Next, compounds containing radioactive, toxic, or precious metal elements were excluded because of their lack of suitability for large-scale commercialization. Among the 398 materials that are elemental sustainable, our target materials should exhibit high capacity to achieve effective Na compensation with minimal addition, leaving only 52 compounds with theoretical capacities of >400 mAh g^−1^, of which the average desodiation voltages were calculated by DFT. The voltages were calculated either based on conversion reactions, for materials that do not exhibit an obvious host structure, or based on hypothetical topotactic Na deintercalation reactions.

The distribution of the 52 compounds identified as potential sacrificial additives is summarized in Fig. 1C. The screened materials are first grouped by their anion chemistry: intermetallics, nitrides, sulfides, phosphides, and oxides, which generally fall into different voltages range. For examples, nitride, alloys, and phosphide generally show very low voltage (<1.5 V); sulfides decompose at higher voltages around 2 V. The only fluoride appears in our screening results is a hypothetical compound Na_2_HF_4_, in which Na ions are octahedrally coordinated by F^−^, indicating a high energy penalty (~6 V) for removing Na from the structure. Most screened oxide materials have predicted voltages within 2 to 4 V, which is the typical voltage window for most NIBs. Other than Na_2_O_2_ discussed above, Na_14_Mn_2_O_9_ appears as a promising presodiation materials with the highest theoretical capacity of 651.7 mAh g^−1^ at an average potential of 2.77 V. Also, a variety of ternary oxides (Na-M-O) with different Na-to-metal ratio are identified. For instances, Na_6_MO_4_ (M = Zn) forms a hexagonal crystal structure (fig. S1) and exhibits a theoretical capacity of about 602 mAh g^−1^ (*33*). Na_5_MO_4_ (M = Mn, Co, Sb and In) crystallizes in the orthorhombic space group with a theoretical capacity of around 560 to 580 mAh g^−1^ (*34*, *35*). Na_4_MO_4_ (M = Co, Fe, Mn and V) features a triclinic structure and demonstrates theoretical capacities of ~500 mAh g^−1^ (*36*).

We are particularly interested in oxide materials because of their chemical compatibility with common oxide-based cathodes. Among the screened oxides, inspired by successful prelithiation agents like Li_5_FeO_4_, we prioritized ternary Na-M-O phases with high Na-to-TM ratios to maximize sodium reservoir capacity while also considering elemental cost and abundance for practical relevance. Therefore, the Na_4_MO_4_ and Na_5_MO_4_ framework stand out for their ability to accommodate various metal species, suggesting a substantial possibility for designing electrochemically active presodiation additives. Our experimental efforts thus focus on these two families, which leads to the successful synthesis of four representative compounds, including Na_4_MO_4_ (M = Fe, Ti) and Na_5_MO_4_ (M = Fe, Ni). The XRD patterns and Rietveld refinement profiles of the as-synthesized materials are shown in figs. S2 to S5, together with detailed structural parameters in tables S3 to S6. All samples except Na_5_FeO_4_ show high refined purity of above 95 wt %, with only trace amount of Na_2_CO_3_ or NaOH as impurity. For Na_5_FeO_4_, the phase purity of as-synthesized sample is 70.7 wt %, with 15.4 wt % Na_4_FeO_4_, 7.4 wt % Na_2_O_2_, and 6.5 wt % NaOH.

The presodiation performance is evaluated in Na half-cells in Fig. 1 (D and E). Na_5_MO_4_ materials deliver high presodiation capacities at 4.5-V cutoff voltage, reaching 537 and 479 mAh g^−1^ for M = Ni and Fe, respectively. Although the measured capacity of Na_5_FeO_4_ is lower than that of Na_5_NiO_4_ due to its lower purity, the overall presodiation capacity remains substantial. As for the Na_4_MO_4_ family, Na_4_TiO_4_ exhibits a lower first-charge capacity of only 251 mAh g^−1^, which may originates from the strong Ti─O bonds that suppress oxygen redox activity (*37*). In contrast, NFO exhibits a markedly higher first-charge capacity of 451 mAh g^−1^, corresponding to nearly 90% of its theoretical capacity (505.9 mAh g^−1^). This also explains the high presodiation capacity of Na_5_FeO_4_ despite its lower purity as the main impurity, NFO, is itself electrochemically active and contributes to the overall capacity. These preliminary results prove the efficiency and accuracy of our high-throughput screening for identifying promising presodiation additives. In particular, NFO, featuring high purity, high decomposition ratio, low raw-material cost, and simple synthesis route, was selected for in-depth investigation.

### Experimental synthesis and characterization of NFO

**Fig. 2.**
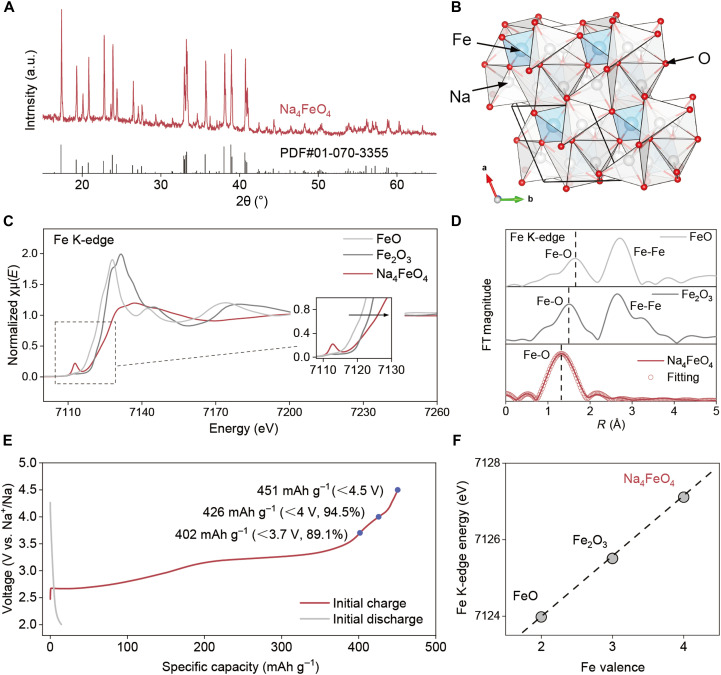
Structural and electrochemical characterization of NFO. XRD patterns (**A**) and crystal structure (**B**) of Na_4_FeO_4_ (NFO). a.u., arbitrary units. (**C**) Fe K-edge x-ray absorption near-edge structure (XANES) spectra of FeO, Fe_2_O_3_, and NFO (inset shows enlarged pre-edge region). (**D**) FT *k*^2^-weighted χ(*k*)-function of the x-ray absorption fine structure (EXAFS) spectra for the Fe K-edge and EXAFS fits for NFO. (**E**) First charge-discharge profile of NFO electrode at 0.1 C (1C = 450 mA g^−1^). (**F**) Linear fit of Fe valence versus Fe K-edge energy position.

The powder XRD pattern of the as-prepared NFO is shown in Fig. 2A, indicating a pure NFO phase in good accordance with the standard card (PDF#01-070-3355). According to the XRD results, NFO crystallizes in a triclinic P̅1 unit cell (Fig. 2B) with two distinct tetrahedral Fe sites and eight Na sites. Half of the Na ions are tetrahedrally coordinated, while the others exhibit fivefold coordination in distorted NaO_5_ pyramids. Each Na polyhedron is edge sharing or corner sharing with the two Fe tetrahedra, constituting a three-dimensional network. Figure S6 displays the scanning electron microscopy (SEM) image and energy-dispersive spectroscopy (EDS) mapping of NFO, indicating a uniform elemental distribution of Na, Fe, and O. Thermogravimetric analysis curve in fig. S7 reveals that NFO exhibit good thermal stability above 800°C.

The stoichiometry of the as-synthesized sample was verified by inductively coupled plasma (ICP) analysis (table S7), indicating that Fe is in tetravalent oxidation state, which is rarely achieved via solid-state methods (*38*, *39*). Therefore, XAS was performed to verify the oxidation state of Fe. As shown in Fig. 2C, the Fe K-edge -ray absorption near-edge structure spectra (XANES) of NFO present a well-defined pre-edge peak, which is induced by the 1s-3d electron transition arising from tetrahedral coordination (*40*). The position of the absorption edge of NFO exhibits a marked shifted toward a higher energy compared to those of Fe(II)O and Fe(III)_2_O_3_ references, providing direct evidence for the presence of tetravalent Fe. Moreover, as Na_5_FeO_4_ also features tetrahedral local Fe environments, it serves as an alternative reference for Fe^3+^. The distinct shift of the absorption edge to higher energy observed for NFO relative to that of Na_5_FeO_4_ (fig. S8) further supports that the Fe oxidation states in NFO exceeds +3. Figure 2D shows the *k*^2^-weighted Fourier-transformed (FT) x-ray absorption fine structure (EXAFS) spectra, in which a continuously decrease in Fe─O bond length is observed, i.e., from 1.65 Å in Fe(II)O to 1.50 Å in Fe(III)_2_O_3_ and 1.29 Å in Na_4_Fe(IV)O_4_, indicating an elevation in the valence states of Fe. Linear fit of Fe valence versus Fe K-edge energy position shows that the valence state of Fe in NFO is +4 (Fig. 2F).

The detailed electrochemical property of NFO was further explored in the half-cells with sodium metal as the counter electrode. Figure 2E shows the first galvanostatic charge-discharge profile of NFO cycled at 0.1 C in a voltage range of 2 to 4.5 V. Overall, NFO delivers a specific capacity of 451 mAh g^−1^ during the first charging process, while the discharge capacity is only 15 mAh g^−1^, and no capacity is observed during the subsequent cycles, indicating that the desodiation is highly irreversible. In particular, most of the capacity can be delivered at relatively low voltages, e.g., 402 mAh g^−1^ capacity can be obtained with 3.7-V cutoff voltage, and 426 mAh g^−1^ capacity can be obtained below 4 V, demonstrating superior compatibility with the voltage window of current Na cathodes such as NVP (3.7 V) and layered oxides (4.0 V).

### Electrochemical decomposition mechanism of Na_4_FeO_4_

To investigate the decomposition mechanism of NFO, DFT calculations were first applied to determine the desodiation pathway. When Na is removed from NFO during charging, the desodiation reaction may proceed via either topotactic reactions forming Na_4−*x*_FeO_4_ (0 ≤ *x* ≤ 4) solid solution or via nontopotactic reactions, i.e., decomposing into a different structure with less amount of Na. For decomposition reactions, to identify which phase may form when certain amount of Na is removed, we considered all ground states identified in the Materials Project Na-Fe-O phase diagram as decomposition products (*29*), i.e., FeO, Fe_2_O_3_, Fe_3_O_4_, FeO_2_, NaFeO_2_, Na_2_FeO_3_, and Na_3_FeO_3_, and evaluated all possible decomposition reactions. As the Fe in decomposition products is lower in valence than NFO, the decomposition reactions during charging are balanced with the participation of oxygen species, i.e., direct release of O_2_ gas or the formation of intermediate lattice peroxide species (*41*). As reported by Jung and co-workers (*41*), the reaction potential for the formation of peroxide phase (Na_2_O_2_) is always lower than that of forming peroxide species in the lattice and, therefore, can be taken as a proxy for any peroxide reactions. Overall, with *A* representing possible Fe-containing, ground-state decomposition products and with *C* representing O_2_ gas or Na_2_O_2_, the reaction equation can be presented as follows

$${\text{Na}}_{4-x}{\text{FeO}}_{4}\to a\;A+b\;{\text{Na}}^{+}+b\;e^{-}+C\;\left(O_{2}\;\text{or}\;{\text{Na}}_{2}O_{2}\right)$$ (1)

**Fig. 3.**
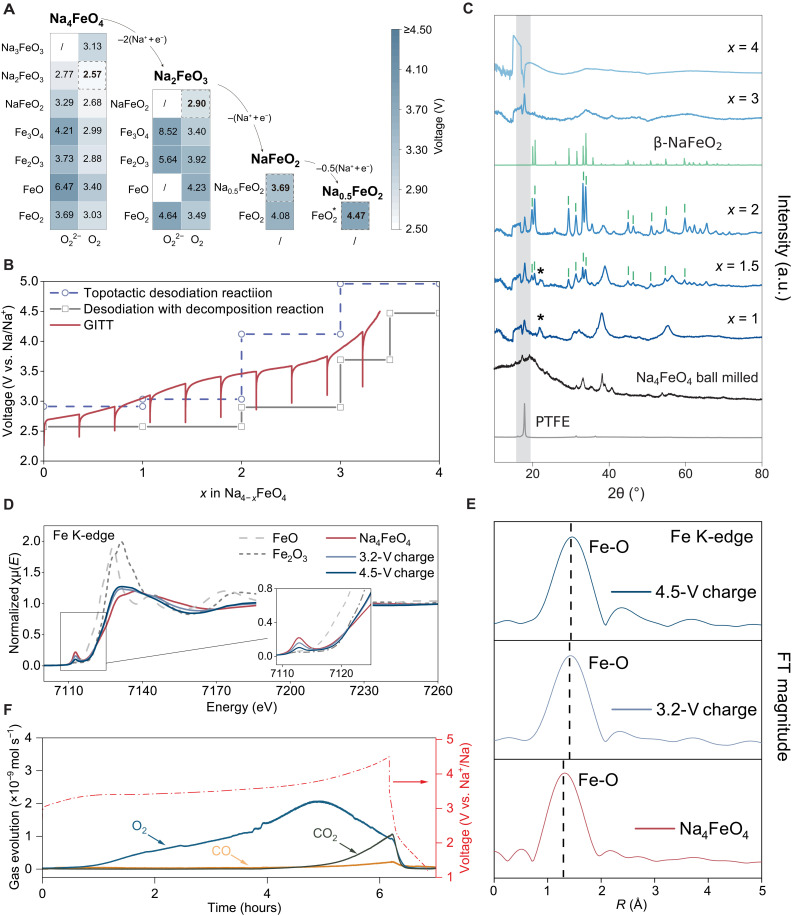
Decomposition mechanism of NFO. (**A**) Predicted voltages for all possible nontopotactic stepwise desodiation reactions: e.g., the first column describes the decomposition of NFO, with seven possible Fe-containing products (row) balanced by oxygen gas or peroxide (column), yielding 14 reactions in total. The lowest-voltage product (Na_2_FeO_3_) is evaluated for the next stepwise reaction. (**B**) Calculated voltage profiles and galvanostatic intermittent titration technique (GITT) results. (**C**) Synchrotron XRD patterns of Na_4−*x*_FeO_4_ (*x* = 1, 1.5, 2, 3, 4) electrode films. Lab XRD pattern of ball-milled NFO powder is shown as reference (black). The shaded area denotes the peak of PTFE binder; the green curve denotes β-NaFeO_2_ phase; asterisks mark an unidentified phase appear in the early stage of decomposition. (**D**) Fe K-edge XANES spectra of FeO, Fe_2_O_3_, and NFO at different state of charge (inset: enlarged pre-edge region). (**E**) FT *k*^2^-weighted χ(*k*)-function of the EXAFS spectra for the Fe K-edge. (**F**) DEMS results of time-resolved evolution rates for O_2_, CO_2_, and CO during initial charging.

By converting the calculated reaction energy to reaction potential versus Na metal (Supplementary Text 2), the predicted voltages for all possible nontopotactic reactions are illustrated in the heatmap shown in Fig. 3A. Each column in Fig. 3A represents a set of stepwise decomposition reactions, and, from a thermodynamic perspective, the overall reaction will proceed along the pathway with the lowest potential for each step. Taking the first column as an example, among all possible reactions, the decomposition of NFO into Na_2_FeO_3_ and O_2_ has the lowest potential of 2.57 V. By analogy, the subsequent decomposition of Na_2_FeO_3_ releases one Na^+^ at 2.9 V to form α-NaFeO_2_ and O_2_. It is reported that the structure of α-NaFeO_2_ can sustain 0.5 Na^+^ deintercalation, forming Jahn-Teller distorted Na_0.5_FeO_2_, of which the average voltage is calculated to be 3.69 V. Further charging of Na_0.5_FeO_2_ toward 4.5 V triggers irreversible structural changes caused by Fe migration (*42*). Although the 4.5-V structure are not fully characterized, the upper voltage limit of the last 0.5 Na^+^ release can be estimated by topotactic Na extraction (*43*, *44*), which is calculated to be 4.47 V. The overall calculated voltage profile of NFO decomposition is summarized in Fig. 3B.

For the topotactic reaction, structures for Na_4−*x*_FeO_4_ (*x* = 0.5, 1, 2, 3, 4) with various Na configurations were calculated (Supplementary Text 2 and fig. S10) (*45*), and the voltage profile constructed by the lowest-energy configurations is also shown with the dashed line in Fig. 3B. At each step, the decomposition reactions are lower in voltage than topotactic desodiation, indicating that extracting Na ions from NFO with decomposition reactions is thermodynamically favorable. Figure 3B also displays the experimental galvanostatic intermittent titration technique (GITT) result, in which the relaxed quasi-equilibrium potential was obtained after 20 hours of relaxation after each 2 hours of charging at 0.05C. The relaxed potentials for the extraction of first three Na are in excellent agreement with the predicted voltages, confirming the DFT results, while the voltage of the last Na extraction is lower than the predicted value, which will be discussed in later section.

To further investigate the decomposition products, ex situ synchrotron XRD was performed for Na_4−*x*_FeO_4_ at various charge state (*x* = 1, 1.5, 2, 3, 4). As shown in Fig. 3C, the pristine materials show broad diffraction peaks due to the ball milling process used for electrode preparation. When *x* = 1, the diffraction peaks of NFO can still be observed but becomes even more broader, suggesting reduced crystallinity during the charging, likely due to lattice strain or phase transitions associated with desodiation. Besides, an additional peak appears at 2θ ≈ 22°, indicating decomposition reactions occur but the product cannot be indexed, probably due to small particle size or amorphization, as often reported for the decomposition of sacrificial additives (*46*). DFT calculations suggest that the decomposition product at this stage might be Na_2_FeO_3_, which is a ground-state structure in the Materials Project phase diagram but has not been experimentally observed. When further desodiated, the peaks of both NFO and unindexed phase gradually disappear and β-NaFeO_2_ starts to nucleate, of which the diffraction peaks become prominent when *x* = 2. It is worth noting that the experimentally observed NaFeO_2_ in Fig. 3C is the β-phase with tetrahedrally coordinated Fe^3+^, rather than octahedrally coordinated α-NaFeO_2_ as predicted in Fig. 3A. Although the β-phase is 0.05 eV per atom higher in energy than α-phase, it could be kinetically stabilized by minimizing the change in Fe local environment. The XRD peak intensity ratio of this electrochemically formed β-NaFeO_2_ slightly differs from the reference, presumably due to Na vacancies or lattice defects (Supplementary Text 3 and fig. S16). The extraction of the last Na leads to the decomposition of β-NaFeO_2_ and the formation of an amorphous phase. Our combined theoretical and experimental results demonstrate that the desodiation of NFO is through nontopotactic decomposition reactions, and the exact pathway is determined by both thermodynamic driving force and local structural kinetics. Although the XRD results deviate from DFT for the last Na extraction, the calculated voltage could provide a reasonable upper voltage limit as the exact structure at 4.5 V cannot be identified.

**Fig. 4.**
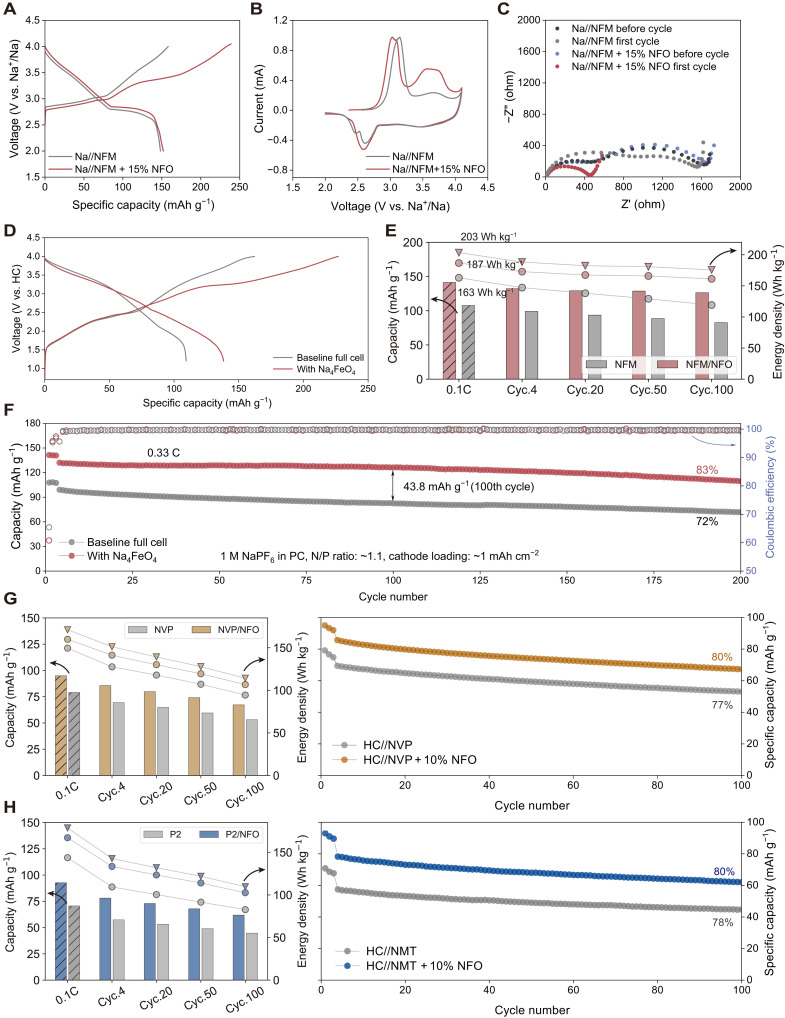
Electrochemical characterization of NFO as presodiation additives. (**A**) Initial charge-discharge profiles of NFM half-cells at 0.1C in the range from 2 to 4 V. (**B**) CV curves at a scan rate of 0.1 mV s^−1^. (**C**) EIS before and after the first cycle. (**D**) Initial charge-discharge profiles of NFM full cells at 0.1C without or with 15 wt % NFO additive. Specific capacity is calculated on the basis of the active materials only. (**E**) Discharge capacity and energy density of NFM full cells without or with 15 wt % NFO at various cycles; the first columns represent data at 0.1C; others at 0.33C. Energy density is calculated on the basis of the total mass of both electrodes including the mass of additives (circles) or excluding it (triangles). (**F**) Full-cell cycling performance at 0.33C in the range from 2 to 4 V. Electrochemical performance of NVP (**G**) and P2-NMT (**H**) full cells without or with 10 wt % NFO additives: discharge capacity and energy density measured at various cycles (left); cycling performance at 0.33C (right). The energy density is calculated with the same approach as described in (E).

To provide more experimental evidence for the decomposition of NFO, ex situ XAS of samples at different state of charge was measured and shown in the Fig. 3D. The reduction in pre-edge peak intensity at 3.2 V provides direct evidence that, upon charging NFO with its distinct tetrahedral Fe coordination, transforms into phases where Fe occupies more centrosymmetric sites. This could likely be assigned to the predicted formation of Na_2_FeO_3_ (Fig. 4A), in which Fe occupies trigonal bipyramidal sites (fig. S17) that feature a reduced pre-edge peak than FeO_4_ tetrahedra (*47*). However, the coexistence of phases containing tetrahedral and octahedral Fe cannot be excluded. At 4.5 V, the pre-edge peak is not fully vanished, consistent with the incomplete decomposition of β-NaFeO_2_ (451 mAh g^−1^ delivered capacity versus 505.9 mAh g^−1^ theoretical value), or the amorphorized decomposition product still exhibits noncentrosymmetric Fe local structure. Also, there is an apparent shift of the absorption edge toward the lower-energy region, confirming that the decomposition process is not compensated by Fe oxidation but oxygen release, consistent with our DFT results. Moreover, the *k*^2^-weighted FT EXAFS spectra are shown in Fig. 3E. The increase in Fe─O bond length also indicates the reduction in the valence states of Fe.

The desodiation process of NFO was also monitored using DEMS. Figure 3F depicts the correlation between Na extraction and gas release, while the amount of generated O_2_ is quantified in fig. S18. The initial charging exhibits a relatively low rate of O_2_ release, which substantially increases after 2 hours of charging, indicating that the Fe^4+^ might be reduced to Fe^3+^; therefore, more oxygen redox is required for charge compensation, which is consistent with the formation of NaFe^3+^O_2_ predicted by DFT. Subsequently, the oxygen evolution is still observed, but the rate is declined, indicating that further Na extraction from β-NaFeO_2_ may also involves certain oxygen redox (*48*). In addition, the emergence of CO_2_ and the trace release of CO are observed at high voltages, possibly due to the slight decomposition of the electrolyte at high potential caused by the generation of oxygen. Therefore, according to the combined DFT, XRD, XAS, and DEMS evidence, the desodiation of NFO proceeds along stepwise decomposition reactions, forming an amorphous oxide phase with Fe^3+^ rather than high-valent metal species. In practical cell manufacturing, the generated gaseous species can be released in a controlled manner (e.g., via degassing of pouch cells) after the formation cycles, making NFO a promising cathode additive for NIBs.

### Electrochemical performance of NFO in Na-ion full cells

The electrochemical performance of NFO as presodiation additives were first tested in Na metal half-cells with NFM as cathodes. The initial charge-discharge curves without and with 15 wt % NFO are shown in Fig. 4A, which exhibit initial charge capacity of 148 and 239 mAh g^−1^, respectively. As Na metal is an infinite sodium source, there is almost no difference in discharge capacity. The cyclic voltammetry (CV) curves (Fig. 4B) of pristine half-cell display two oxidation peaks at 3.14 and 3.68 V, whereas the half-cell with NFO shows two additional oxidation peaks at 3.04 V and 3.57 V, corresponding to the decomposition reaction of NFO. Electrochemical impedance spectroscopy (EIS) of the Na||NFM and Na||NFM + 15 wt % NFO cells was shown in Fig. 4C. Before cycling, the impedance of electrodes with and without NFO shows no obvious difference. After the initial charge-discharge cycle, the electrode containing NFO exhibits a substantially lower impedance compared to the additive-free electrode, likely due to the formation of gas pores within the electrode film that enhances infiltration of electrolyte and reduces the tortuosity for Na transport. This porous electrode morphology can be observed by the cross-sectional SEM of the electrode after the formation cycle (figs. S19 and S20).

To demonstrate the practical application of NFO, the full-cell performance based on HC anodes and NFM cathodes was investigated. The electrochemical properties of HC were first tested and shown in fig. S21. It has an ICE of 73% at 0.1 C with a reversible specific capacity of ≈280 mAh g^−1^. The initial charge-discharge curves of the full cells are shown in Fig. 4D. The pristine NFM full cell consumes a high fraction of active sodium irreversibly during the first cycle: At 0.1C, the initial discharge capacity is only 109 mAh g^−1^, leading to an ICE of 67.6%. In contrast, the initial discharge-specific capacities of full cells with 15 wt % NFO additives increased markedly to 141 mAh g^−1^, leading to a notable increase of energy density. With the addition of NFO, Fig. 4E shows that gravimetric energy density (calculated on the total mass of both electrodes including the mass of additives) is increased from 163 to 187 Wh kg^−1^ at 0.1C. If excluding the mass of additive, the energy density reaches 203 Wh kg^−1^, corresponding to a 24.5% increase. Because gaseous products from NFO decomposition can be evacuated after formation, the practical energy density is expected to lie between these two bounds. Moreover, the cycle stability of the full cell is also enhanced. After 200 cycles, the specific capacity of the NFM full cell containing NFO remained at 110 mAh g^−1^, with a capacity retention of 83% (Fig. 4, E and F), compared to 72% of the pristine full cell. This agrees well with the consistently higher coulombic efficiency (CE) for full cells incorporating NFO compared to that without NFO (fig. S22). The improved capacity retention also correlates with interfacial stability. The electrochemical impedance in fig. S23 only shows a slight increase after 30 cycles. Also, as evidenced by the x-ray photoelectron spectroscopy data in figs. S24 and S25, both the cathode-electrolyte interphase (CEI) and solid-electrolyte interphase (SEI) in full cells with NFO remain stable upon cycling. Notably, no dissolute Fe was detected in either CEI or SEI (figs. S26 and S27), further confirming the robustness of the interfaces.

Full cells with different loading of NFO (5 and 10 wt %) were also tested (figs. S28 and S29). The initial discharge capacities of full cells with 5 and 10 wt % NFO additives are 113 and 123 mAh g^−1^, with capacity retention of 84 and 80% after 200 cycles, respectively. Therefore, the addition of NFO not only increases the full-cell energy density (fig. S30) but also shows no negative impact for the subsequent cycling performance. On the contrary, it could enhance full-cell stability by compensating for subsequent sodium loss during cycling. In addition, the full-cell performance of other identified materials, i.e., Na_5_NiO_4_ and Na_5_FeO_4_, are shown in figs. S31 and S32, both demonstrating effective presodiation performance.

To evaluate the universality of NFO as presodiation additives, we systematically assessed the full-cell performance with diverse cathode materials. For NVP cathodes, as shown in Fig. 4G, full cell with 10 wt % NFO additive shows increased initial discharge capacity (94.9 mAh g^−1^) compared to the pristine cell (79.1 mAh g^−1^), demonstrating the effective decomposition of NFO at a low cutoff voltage of 3.7 V (fig. S33). The energy density is also increased from 140.7 to 165.5 Wh kg^−1^. After 100 cycles, it maintains a capacity retention of 80%, while the capacity retention of full cells without NFO is 77%. In addition, the full cell containing NFO shows a stable and slightly higher CE because of the additional NFO (fig. S34). NVP full cells with 3 wt % NFO additive also exhibit improved electrochemical performance (figs. S35 to S37). Similarly, the full-cell performance for Na-deficient P2-Na_0.67_Ni_0.33_Mn_0.33_Ti_0.33_O_2_ (NMT) was also evaluated (figs. S39 and S40). The HC||NMT + 10 wt % full cell demonstrate an increased initial discharge capacity from 70.6 to 92.7 mAh g^−1^ (Fig. 4H). After 100 cycles, the capacity retention of HC||NMT + 10 wt % cell reaches 80%, maintaining a specific capacity of 61.9 mAh g^−1^, which is notably higher than 44.7 mAh g^−1^ of the NFO-free full cell. Moreover, we also test the electrochemical performance of NFO paired with tunnel-type Na_0.44_MnO_2_ cathode (fig. S41), whose inherent Na deficiency makes it a particularly relevant class of materials for presodiation. As shown in fig. S42, the incorporation of 15 wt % NFO substantially increases the initial discharge capacity from 38.5 to 89.0 mAh g^−1^. These results demonstrate that NFO as an effective presodiation additive exhibits broad compatibility with most Na-ion cathode materials, enabling notable improvement in full-cell energy density and cycle stability.

## DISCUSSION

**Fig. 5.**
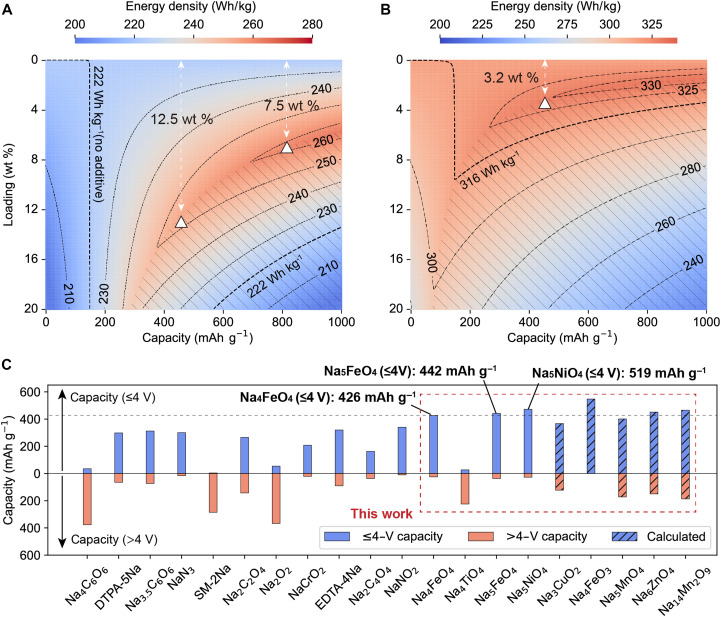
The role of presodiation additives in NIBs. Contour map depicting the full-cell energy density as a function of the mass loading and the capacity of presodiation/lithiation additives in NIBs (**A**) and LIBs (**B**). The thick dashed curves define baseline energy density without additives; the shaded region indicates that SEI loss is fully compensated by additives. Triangles marks the maximum attainable energy density at additive capacity of 450 or 800 mAh g^−1^. (**C**) Comparison of presodiation capacities below and above 4 V for various reported additives—Na_4_FeO_4_, Na_4_TiO_4_, Na_5_FeO_4_, and Na_5_NiO_4_—and several other predicted candidates in this work.

The incorporation of cathode presodiation additives presents a compelling strategy to enhance full-cell energy density and cycle stability. However, their mass loading requires critical optimization to balance the electrochemical performance gains against potential degradation risks. Taking a typical O3||HC full-cell configuration as an example, Fig. 5A shows the calculated energy density (based on the mass of both electrodes, details in Supplementary Text 4) as a function of the mass loading and the capacity of presodiation additives. The two thick dashed curves in Fig. 5A define the baseline energy density without additives; thus, the enclosed area indicates where incorporating additives enhance energy density. When the amount of supplemental Na ions is less than the irreversible loss from SEI formation, adding more additives consistently increases full-cell energy density, e.g., rising from a baseline of 222 Wh kg^−1^ to a maximum of ~260 Wh kg^−1^, with a substantial energy density gain of ~18%. However, when SEI losses are fully compensated, as shown by the shaded region, further increasing the additive loading introduces excess Na ions that beyond the cathode intercalation capability, thus reducing the overall energy density. When such an oversodiation occurs, the excess Na can be either accommodated by the anode under sufficient Negative-to-Positive capacity (N/P) ratio conditions, forming a sodium reserve that mitigates cycle-induced capacity fade, or under low N/P ratio conditions, in which sodium metal plating could be triggered, which accelerates the cell degradation and raises safety concerns. As a reference, for a practical O3||HC full cell with a N/P ratio of 1.1 (based on the reversible capacity), the maximum loading of NFO is calculated to be 13.99 wt % (Supplementary Text 5 and fig. S43).

Figure 5A essentially demonstrates the improvement margin for full-cell energy density with the incorporation of additives with various capacity. For instances, for presodiation additives with a capacity of 450 mAh g^−1^, e.g., NFO, the maximum energy density of 253 Wh kg^−1^ can be reached with a loading of 12.5 wt %; for a hypothetical additive with 800 mAh g^−1^ capacity, the maximum energy density increased to 259 Wh kg^−1^ with 7.5 wt % loading. The improvement margin is more pronounced for NIBs using Na-deficient P2 cathodes because the preexisting Na vacancies in these cathodes can accommodate additional Na ions, potentially leading to an ICE exceeding 100%. As shown in fig. S44, for the hypothetical additive with a capacity of 800 mAh g^−1^ and a loading of 12.3 wt %, the full-cell energy density could reach ~243 Wh kg^−1^, which matches that of O3-type cells, in sharp contrast to P2 full cells without presodiation (~157 Wh kg^−1^). In this sense, presodiation is of more importance for NIBs than for LIBs. As shown in Fig. 5B, the calculated energy density of LiFePO_4_||graphite full cell exhibits a much narrower improvement margin, e.g., adding 3.2 wt % of prelithiation additives with 450 mAh g^−1^ capacity increases the energy density from a baseline of 316 Wh kg^−1^ to the maximum of 329 Wh kg^−1^, corresponding to an energy density gain of only 4.1%. This performance gap derives fundamentally from the inferior ICE of HC compared to graphite, which exacerbates initial energy losses and mandates elevated additive loading in NIBs.

Besides, presodiation additives may also enhance Na^+^ transport kinetics primarily from a macroscopic pore–forming effect, where additive decomposition creates micro-cracks (figs. S19 and S20) that increase electrode porosity and reduce ionic tortuosity. At the microscopic scale, the controlled release of sodium and oxygen during the formation cycles may indirectly regulate the formation of interphases, thereby maintaining low charge-transfer resistance (*49*, *50*). Nevertheless, increased additive loading may raise degradation risks as higher loadings can largely alter electrode architecture by exposing fresh surfaces that might induce parasitic reactions during cycling. The increased porosity may weaken the electronic percolation network and compromise the mechanical robustness of the electrode. Therefore, developing high-capacity presodiation additives to minimize mass loading is essential for simultaneously enhancing the energy density and the cycle life for NIBs. Figure S45 and table S8 summarize the capacity and average decomposition voltage for presodiation materials developed in this work and reported in literature, where Na_4_FeO_4_, Na_5_FeO_4_, and Na_5_NiO_4_ not only deliver high irreversible capacities but also decompose at substantially lower average voltages, demonstrating their promising potential as efficient presodiation agents.

Under the 4-V cutoff for NIBs, Fig. 5C compares the achievable presodiation capacity (<4 V) of reported additives alongside selected candidates identified in our high-throughput screening. As numerous existing additives exhibit high decomposition voltages, limiting their utilization efficiency within the 2- to 4-V operational window, we have identified several ternary sodium transition metal oxides demonstrating both high capacity and low decomposition potentials. While Na_4_FeO_4_, Na_5_FeO_4_, and Na_5_NiO_4_ represent a benchmark performance of 426, 441, and 519 mAh g^−1^ at a cutoff voltage of 4 V, other compounds such as Na_4_FeO_3_, Na_6_ZnO_4_, and Na_14_Mn_2_O_9_ also emerge as promising candidates worth exploring in future research. Besides discovering previously unidentified materials, several approaches can also be explored to improve the practical capacities of the as-synthesized materials, which are lower than theoretical values (table S9), likely due to sluggish Na^+^ diffusion or poor electronic conductivity. These include (i) nanostructuring to shorten diffusion paths (*26*, *51*, *52*), (ii) carbon coating to enhance electron transport (*53*, *54*), (iii) using highly conductive carbon additives (*24*), and (iv) controlled charge protocols (e.g., lower rate and intermittent rest) to allow more complete decomposition.

In conclusion, we demonstrate a combined theoretical and experimental investigation of Na_4_MO_4_ (M = Ti, Fe) and Na_5_MO_4_ (M = Ni, Fe) structural families as cathode presodiation additives, which show high irreversible capacities above 450 mAh g^−1^ with low decomposition voltages. With the addition of NFO, substantial increases in both full-cell specific capacity and energy density can be achieved with various cathode chemistries. Besides, our high-throughput screening provides various potential presodiation additives, such as Na_14_Mn_2_O_9_, Na_6_ZnO_4_, and Na_4_FeO_3_, demonstrating presodiation additives as a strategic research frontier that may facilitate the practical applications of NIBs.

## MATERIALS AND METHODS

### Synthesis

NFO material was synthesized by a solid-state reaction method from stoichiometric mixtures of Na_2_O_2_ and Fe_2_O_3_. The mixtures were ground and pressed into pellets under 20 MPa, and, then, the pellets were calcined in a sealed corundum crucible at 450°C in Ar for 16 hours, cooled to room temperature, and stored in an argon-filled glove box. Na_4_TiO_4_ (Na_5_FeO_4_) was synthesized from stoichiometric mixtures of Na_2_O and TiO_2_ (Fe_2_O_3_). The mixtures were ground and pressed into pellets under 20 MPa, and, then, the pellets were heated to 500°C (420°C) in Ar for 5 hours (24 hours). Na_5_NiO_4_ was synthesized from stoichiometric mixtures of Na_2_O and NaNiO_2_. NaNiO_2_ was synthesized from stoichiometric mixtures of Na_2_O and NiO at 650°C in O_2_ for 14 hours. Then, the mixtures of Na_2_O and NaNiO_2_ were ground and pressed into pellets under 20 MPa and calcined in a sealed corundum crucible at 550°C in Ar for 48 hours. P2-NMT cathode material was synthesized by a solid-state reaction method (*55*) from stoichiometric mixtures of Na_2_CO_3_, NiO, Mn_2_O_3_, and TiO_2_. The mixtures were ground and pressed into pellets under 20 MPa, and, then, the pellets were calcined at 950°C in air for 15 hours, cooled to room temperature, and stored in an argon-filled glove box until use. Na_0.44_MnO_2_ was synthesized by a solid-state reaction method from stoichiometric mixtures of Na_2_CO_3_ and Mn_2_O_3_. The mixtures were ground and pressed into pellets under 20 MPa, and, then, the pellets were calcined in a corundum crucible at 950°C in air for 12 hours, cooled to room temperature, and stored in an argon-filled glove box.

### Electrochemistry

Cathode active material was mixed with Ketjen Black and polytetrafluoroethylene (PTFE; beads, Sigma-Aldrich) at a weight ratio of 85:10:5. The active material and Ketjen Black were first hand mixed in a mortar for 20 min, and, then, PTFE was added, followed by mixing and pressing for another 10 min. The mixture was then transferred onto a flat and smooth stainless-steel plate and further pressed and rolled using a stainless-steel stick. The obtained self-standing sheets were then punched into 12-mm disk electrodes for subsequent characterizations. For electrode with presodiation additives, NFO with mass loading of 5, 10, or 15 wt % of the mixture was added before rolling into electrode disks, respectively. For anode, the HC anode electrodes were prepared by mixing HC (85 wt %) with carbon black (Super P, 8 wt %) and polyvinylidene difluoride (7 wt %) in *N*-methyl pyrrolidone to form a homogenous slurry. The slurry was spread onto a carbon-coated aluminum current collector and dried at 110°C for 12 hours in dynamic vacuum. The resulting electrode sheet was then punched into 14-mm disk electrodes for subsequent characterizations.

The cells were assembled in an argon-filled glove box using 1 M NaPF_6_ dissolved in propylene carbonate (PC) with 2 vol % fluoroethylene carbonate as the electrolytes and glass fiber (GF/D) as the separators. The amount of electrolyte is 150 μl in both half- and full-cell configuration. The reversible N/P ratio (based on the reversible capacity of active materials) remains strictly constant at ~1.45 for cells with and without presodiation, while the first-charge N/P ratio (based on the first-charge capacity of active materials and additive) varies between 1.1 and 1.45 depending on the various mass loading of NFO. The assembled cells were galvanostatically cycled at 25°C using a program-controlled test system (LAND CT2001A). CV measurements were carried out on a Princeton instruments testing system. EIS experiments were conducted in the frequency range of 0.01 to 100 kHz with a Princeton electrochemical analyzer by applying an ac voltage of 10-mV amplitude. GITT measurement was programmed by supplying a constant current flux of 0.05 C for 2 hours followed by an open-circuit stand for 20 hours, respectively.

### Characterization

The crystalline structures of the as-prepared products were investigated by using powder XRD with Cu Kα radiation (λ = 1.5416 Å) at a scanning rate of 2° min^−1^. Rietveld refinement was performed using the GSAS-II software package (*56*, *57*). The morphologies and structures were characterized by SEM (Tescan, MIRAS3 FE-SEM) with EDS. To study the change of morphology, the electrodes were disassembled in a glove box after being cycled. Then, the electrodes were washed with PC to remove the electrolyte from the electrode surface. Thermal decomposition of NFO was investigated using a thermogravimetric analyzer at a heating rate of 10°C min^−1^ under Ar flow from room temperature to 900°C, respectively. The elemental compositions of NFO were verified by ICP. Synchrotron powder XRD patterns with a wavelength of λ = 0.4834 Å were collected at the beamline BL11B (PT-506467) of the Shanghai Synchrotron Radiation Facility. All ex situ synchrotron x-ray diffraction (sXRD) samples were prepared and measured as electrodes, while the pristine one as powder. Hard XAS Fe K-edge XAS measurements were performed at Beamline BL17UM (PT-506468) at Shanghai Synchrotron Radiation Facility. The sample was wrapped with Kapton tape and transferred from a glove box filled with argon gas to a transport container to minimize the potential exposure to air. The data were treated using the Athena package for energy calibration and normalization (*58*), and the edge positions were determined from the maximum of the first derivative of the normalized spectra. The DEMS setup consists of a commercial quadrupole mass spectrometer (Hiden HPR-20) equipped with turbo molecular pump (Pfeiffer Vacuum) and rotary pump (Edwards Vacuum), a homemade cold trap, an electrochemical cell (EL CELL GmbH), and a digital mass flowmeter controller (MFC), which can be found elsewhere (*59*). The Ag wire was used as the reference electrode. The Ar was used as carrier gas, and the flow rate was 0.3 ml min^−1^ controlled by the MFC.

### Computation

All calculations in this study were performed with DFT using the Vienna Ab initio Simulation Package (VASP) package. Projector augmented-wave potentials with a kinetic energy cutoff of 520 eV and the exchange-correlation form in the Perdew-Burke-Ernzerhof generalized gradient approximation were used with spin polarization and rotationally invariant scheme of Hubbard *U* correction (*60*). All INCAR parameters including the values of *U*_eff_ (5.3 eV for Fe) were benchmarked by the Materials Project and are documented in MPRelaxSet in Python Materials Genomics (pymatgen) (*45*). The convergence criteria were set as 10^−5^ eV for electronic iterations and 0.02 eV/Å for ionic iterations, with a *k*-point grid with a density of at least 1200/(number of atoms). Gaussian smearing with a width of 0.05 eV was implemented to enhance the convergence of electronic minimization. DFT-D3 method was used to incorporate van der Waals interaction (*61*).

## Supplementary Materials

### The PDF file includes:

Tables S1 to S9 Figs. S1 to S45 Supplementary Text 1 to 5 Legend for data S1 References
