# Supplementary material for: Discovering high-efficiency cathode presodiation additives for Na-ion batteries via high-throughput screening
Source: Sci Adv. 2026 May 15;12(20):eaed4045. doi: 10.1126/sciadv.aed4045 (PMC13178537; doi:10.1126/sciadv.aed4045)
Supplement: Supplementary file 1 — Tables S1 to S9 Figs. S1 to S45 Supplementary Text 1 to 5 Legend for data S1 References [file sciadv.aed4045_sm.pdf]

Science Advances
AAAS

Supplementary Materials for

Discovering high-efficiency cathode presodiation additives for Na-ion batteries via high-throughput screening

Di Wu *et al.*

Corresponding author: Jingyang Wang, jy_wang@nju.edu.cn; Zhengyan Lun, zylun@ucas.ac.cn; Yan Jin, yanjin@nju.edu.cn; Jia Zhu, jiazhu@nju.edu.cn

Sci. Adv. **12**, eaed4045 (2026)
DOI: 10.1126/sciadv.aed4045

The PDF file includes:

Tables S1 to S9
Figs. S1 to S45
Supplementary Text 1 to 5
Legend for data S1
References

Other Supplementary Material for this manuscript includes the following:

Data S1

Table S1. High-throughput screening results for potential presodiation additives

	mpid	Chemical formula	E_{hull} (eV /atom)	Band gap (eV)	Capacity (mAh/g)	Voltage (V vs. Na/Na^+)
1	mp-1019888	KNa2BN2	0	1.82	432.60	0.38
2	mp-616702	Na10Co4O9	0	1.36	439.61	2.93
3	mp-36165	Na10Zn4O9	0	1.49	421.70	2.82
4	mp-540734	Na14Mn2O9	0	0.89	651.70	2.77
5	mp-37458	Na2HF4	0	1.29	435.84	6.10
6	mp-565246	Na2MnO2	0	0.27	403.26	2.25
7	mp-2340	Na2O2	0	1.74	687.37	3.21
8	mp-5122	Na3AlP2	0	1.35	509.19	1.19
9	mp-985586	Na3BrO	0	1.88	487.65	3.55
10	mp-31584	Na3CoO3	0	1.77	457.08	3.42
11	mp-561742	Na3CoO3	0.00015625	1.75	457.08	3.44
12	mp-755401	Na3CuO2	0	1.37	488.71	2.54
13	mp-761827	Na3CuO3	0.0043196	1.17	445.40	3.20
14	mp-28556	Na3CuO3	0	0.78	445.40	3.17
15	mp-778416	Na3FeO4	0.00838883	0.44	425.82	3.67
16	mp-764457	Na3FeO4	0.00248449	0.32	425.82	4.41
17	mp-781774	Na3NiO3	0.00153783	1.68	457.70	2.73
18	mp-1598	Na3P	0	0.40	804.46	0.67
19	mp-7956	Na3Sb	0	0.40	421.54	0.74
20	mp-18762	Na4CoO3	0	1.27	538.99	2.55
21	mp-561627	Na4CoO3	7.92E-05	1.11	538.99	2.49
22	mp-31593	Na4CoO4	0	1.37	498.86	3.51
23	mp-19026	Na4FeO3	0	1.39	547.49	2.54
24	mp-19022	Na4FeO4	0	0.35	506.13	3.76
25	mp-629499	Na4FeO4	0	0.33	506.13	3.76
26	mp-853250	Na4MnO4	0.00321562	0.65	508.31	3.27

27	mp-29871	Na4SeO5	0	1.33	427.23	4.50
28	mp-28261	Na4SnO3	0	1.89	414.43	2.55
29	mp-849942	Na4VO4	0	0.99	518.13	3.16
30	mp-641500	Na5Co2O5	0.00170097	1.32	428.37	3.38
31	mp-776861	Na5CoHO4	0	1.18	560.93	3.10
32	mp-776641	Na5CoO4	0.00433576	1.65	563.31	3.31
33	mp-31522	Na5CoO4	0.00011069	1.45	563.31	2.95
34	mp-776650	Na5CoO4	0.00830812	1.52	563.31	3.28
35	mp-757878	Na5Cu(HO2)2	0	1.49	548.04	2.73
36	mp-11147	Na5CuSO2	0	1.52	552.44	2.63
37	mp-762875	Na5FeHO4	0	1.23	568.28	2.88
38	mp-556086	Na5FeS4	0.00190114	0.80	448.08	2.20
39	mp-4172	Na5GeP3	0	1.06	477.70	0.93
40	mp-14493	Na5InO4	0	1.89	456.15	2.75
41	mp-32013	Na5MnO4	0.00276655	1.44	572.93	3.02
42	mp-759876	Na5SbO5	0.00153751	1.64	423.11	3.32
43	mp-5929	Na5SiP3	0	1.35	567.90	1.06
44	mp-18317	Na5SnP3	0	0.94	410.31	1.00
45	mp-5055	Na6MnS4	0	1.40	500.72	2.26
46	mp-7664	Na6ZnO4	0	1.53	601.47	3.23
47	mp-761931	Na8SnO6	0.0048251	1.06	537.85	2.64
48	mp-7440	NaP	0	0.87	496.63	1.03
49	mp-2400	NaS	0	1.23	486.79	1.98
50	mp-409	NaS	0.00252897	0.87	486.79	1.96
51	mp-2402	NaSi	0	1.13	524.72	0.20
52	mp-555936	RbNa7(CoO3)2	0	1.02	407.60	2.63

Table S2. Additional potential presodiation additives under an extended band gap filter (0 – 1.9 eV)

	mpid	Chemical formula	E_{hull} (eV /atom)	Band gap (eV)	Capacity (mAh/g)
1	mp-974920	Na	0.00259984	0	1165.76
2	mp-974558	Na	0.00269365	0	1165.76
3	mp-127	Na	0.00010567	0	1165.76
4	mp-10172	Na	0	0	1165.76
5	mp-973198	Na	0.00023138	0	1165.76
6	mp-982370	Na	0.00301655	0	1165.76
7	mp-1901	NaO2	0	0.0034	487.379
8	mp-570786	KNa2	0	0	630.01
9	mp-1029605	Na2MnN2	0	0	415.73
10	mp-19427	Na7(CoO3)2	0.00856467	0.0019	500.55
11	mp-30794	Na15Sn4	0	0	490.43
12	mp-559023	Na24In5O15	0.00384911	0	470.92

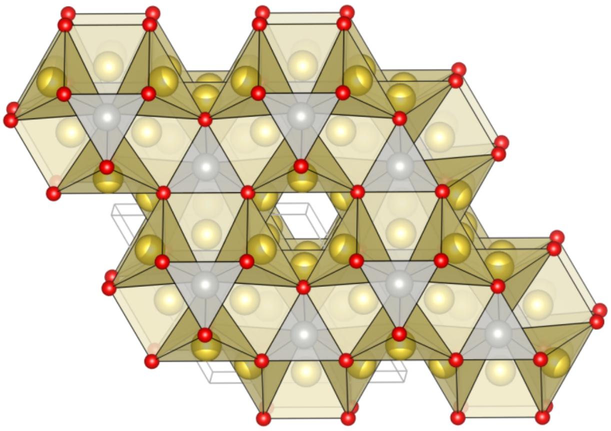

Fig. S1. Crystal structure of Na_6MO_4. The grey, yellow and red spheres represent M, Na and O atoms, respectively.

Supplementary Text 1. Phase purity of as-synthesized materials

Rietveld refinements were performed for all as-synthesized materials, i.e., Na_4FeO_4, Na_4TiO_4, Na_5NiO_4 and Na_5FeO_4, to assess the phase purity using GSAS-II software package, detailed phase purity was summarized as follow:

Na_4MO_4 family: 1) **Na_4FeO_4** was refined using *P-1* space group. It exhibits a high phase purity of 96.3 wt%, with only trace amount of Na_2CO_3 (3.7 wt%) as impurity. 2) **Na_4TiO_4** was refined using *P-1* space group, also exhibiting a high phase purity of 98.0 wt%, and 2.0 wt% Na_2CO_3.

Na_5MO_4 family: 1) **Na_5NiO_4** was refined with *pbca* space group. The refined phase purity was 95.7 wt% with 4.3 wt% NaOH. 2) **Na_5FeO_4** was refined with the same *pbca* space group, however, the phase purity of Na_5FeO_4 is only 70.7 wt%. There exists a substantial amount (15.4 wt%) of Na_4FeO_4, alongside 7.4 wt% Na_2O_2 and 6.5 wt% NaOH. The refined phase fraction indicates that the synthesis reaction could be incomplete, therefore, the optimal synthesis condition for Na_5FeO_4 should be investigated in future studies. However, as Na_4FeO_4 itself is electrochemically active, the presence of this impurity does not substantially compromise the overall presodiation capacity of the sample.

Rietveld refinement profiles and refined structural parameters of all samples were summarized in fig. S2–5 and table S3–6, respectively.

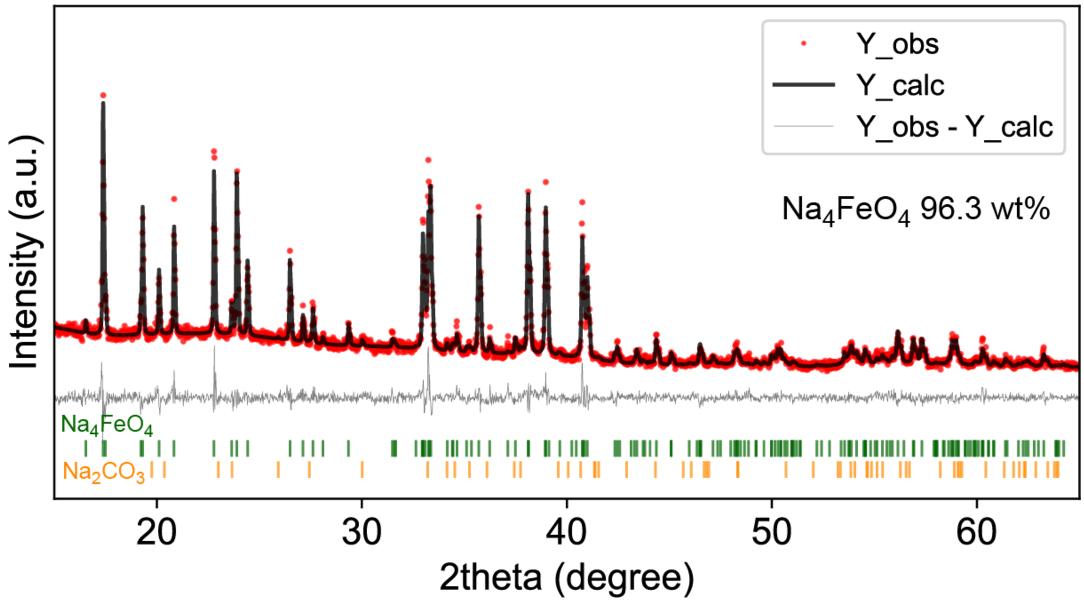

Fig. S2. Rietveld refinement of the powder XRD pattern for Na_4FeO_4. The experimental data (red circles), calculated profile (black line), Bragg positions (ticks), and difference curve (grey line) are shown. The refined main-phase purity is 96.3 wt%.

Table S3. Rietveld refinement structural parameters for Na_4FeO_4.

Structural parameters for Na_4FeO_4, wR = 9.94, GOF = 1.21, Reduced χ^2 = 1.47
Phase fraction: Na_4FeO_4: 96.3 wt%; Na_2CO_3: 3.7 wt%

S.G.	*a* (Å)	*b* (Å)	*c* (Å)	*α (°)*	*β (°)*	*γ (°)*	*V* (Å^3)
P-1	8.477083	5.765625	6.560457	124.67	98.85	101.76	243.266

Atom	site	x	y	z	Occupancy
Fe1	2i	0.250	0.569	0.847	1
Na1	2i	0.569	0.795	0.512	1
Na2	2i	0.976	0.035	0.776	1
Na3	2i	0.824	0.352	0.583	1
Na4	2i	0.349	0.146	0.937	1
O1	2i	0.201	0.164	0.64	1
O2	2i	0.614	0.327	0.302	1
O3	2i	0.688	0.359	0.861	1
O4	2i	0.893	0.237	0.135	1

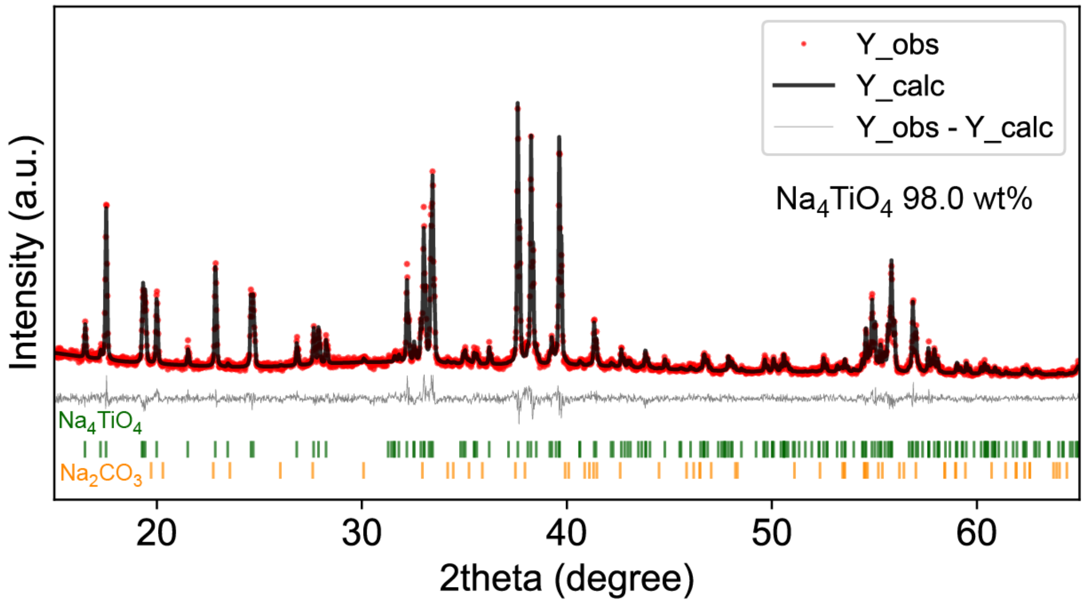

Fig. S3. Rietveld refinement of the powder XRD pattern for Na_4TiO_4. The experimental data (red circles), calculated profile (black line), Bragg positions (ticks), and difference curve (grey line) are shown. The refined main-phase purity is 98.0 wt%.

Table. S4. Rietveld refinement structural parameters for Na_4TiO_4.

Structural parameters for Na_4TiO_4, wR = 11.78, GOF = 1.14, Reduced χ^2 = 1.31
Phase fraction: Na_4FeO_4: 98.0 wt%; Na_2CO_3: 2.0 wt%

S.G.	*a* (Å)	*b* (Å)	*c* (Å)	*α (°)*	*β (°)*	*γ (°)*	*V* (Å^3)
P-1	8.780722	5.776431	6.471193	124.21	102.45	95.76	254.879

Atom	site	x	y	z	Occupancy
Ti1	2i	0.739	0.174	0.397	1
Na1	2i	0.064	0.283	0.047	1
Na2	2i	0.104	0.295	0.558	1
Na3	2i	0.315	0.977	0.208	1
Na4	2i	0.512	0.511	0.770	1
O1	2i	0.125	0.800	0.327	1
O2	2i	0.138	0.834	0.805	1
O3	2i	0.437	0.131	0.786	1
O4	2i	0.695	0.530	0.556	1

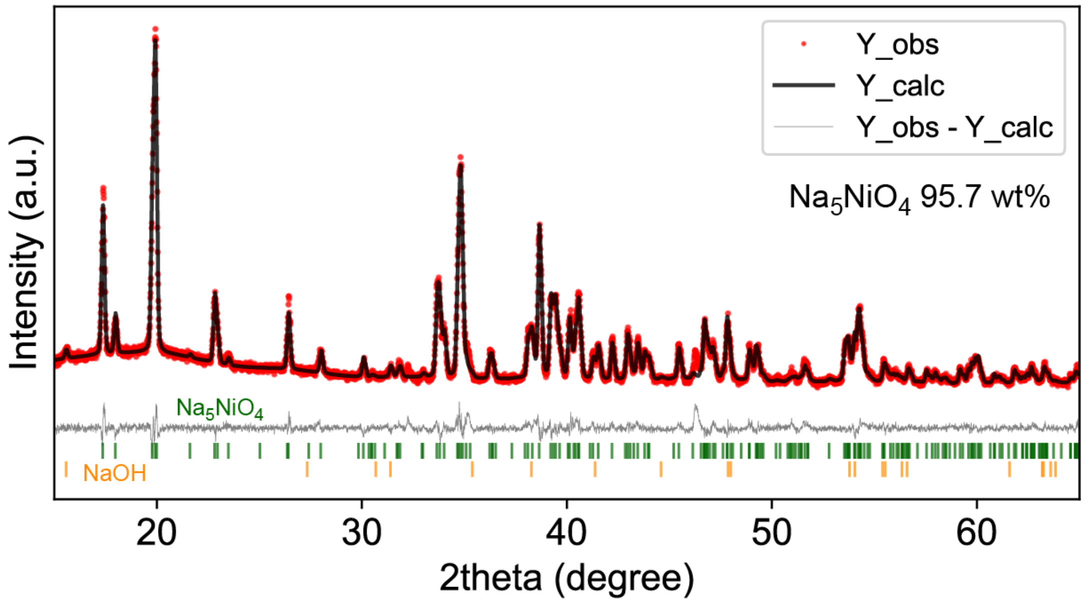

Fig. S4. Rietveld refinement of the powder XRD pattern for Na_5NiO_4. The experimental data (red circles), calculated profile (black line), Bragg positions (ticks), and difference curve (grey line) are shown. The refined main-phase purity is 95.7 wt%.

Table. S5. Rietveld refinement structural parameters for Na_5NiO_4.

Structural parameters for Na_5NiO_4, wR = 4.63, GOF = 3.93, Reduced χ^2 = 15.48
Phase fraction: Na_5NiO_4: 95.7 wt%; NaOH: 4.3 wt%

S.G.	*a* (Å)	*b* (Å)	*c* (Å)	*α (°)*	*β (°)*	*γ (°)*	*V* (Å^3)
Pbca	10.233752	5.947413	17.98249	90	90	90	1094.493

Atom	site	x	y	z	Occupancy
Na1	8c	0.173	0.101	0.829	1
Na2	8c	0.054	0.085	0.434	1
Na3	8c	0.420	0.103	0.752	1
Na4	8c	0.345	0.089	0.501	1
Na5	8c	0.303	0.075	0.140	1
Ni1	8c	0.037	0.176	0.123	1
O1	8c	0.363	0.264	0.375	1
O2	8c	0.127	0.263	0.212	1
O3	8c	0.131	0.197	0.546	1
O4	8c	0.458	0.136	0.620	1

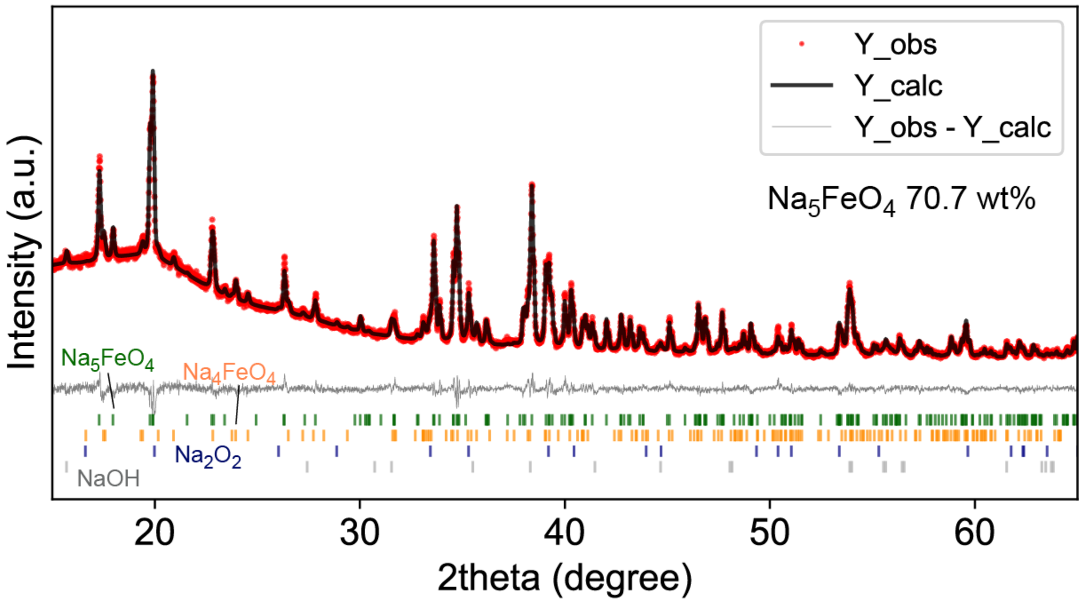

Fig. S5. Rietveld refinement of the powder XRD pattern for Na_5FeO_4. The experimental data (red circles), calculated profile (black line), Bragg positions (ticks), and difference curve (grey line) are shown. The refined main-phase purity is 70.7 wt%, with 15.4 wt% Na_4FeO_4 (orange ticks), 7.4 wt% Na_2O_2 (navy ticks) and 6.5 wt% NaOH (grey ticks) as impurities.

Table. S6. Rietveld refinement structural parameters for Na_5FeO_4.

Structural parameters for Na_5NiO_4, wR = 3.852, GOF = 2.04, Reduced χ^2 = 4.18
Phase fraction: Na_5FeO_4: 70.7 wt%; Na_4FeO_4: 15.4 wt%; Na_2O_2: 7.4 wt%; NaOH: 6.5 wt%

S.G.	*a* (Å)	*b* (Å)	*c* (Å)	*α (°)*	*β (°)*	*γ (°)*	*V* (Å^3)
Pbca	10.33149	5.974357	18.078999	90	90	90	1115.908

Atom	site	x	y	z	Occupancy
Na1	8c	0.171	0.109	0.830	1
Na2	8c	0.061	0.088	0.431	1
Na3	8c	0.423	0.103	0.751	1
Na4	8c	0.341	0.093	0.501	1
Na5	8c	0.303	0.074	0.138	1
Fe1	8c	0.039	0.170	0.124	1
O1	8c	0.352	0.250	0.375	1
O2	8c	0.129	0.276	0.216	1
O3	8c	0.136	0.184	0.543	1
O4	8c	0.447	0.151	0.624	1

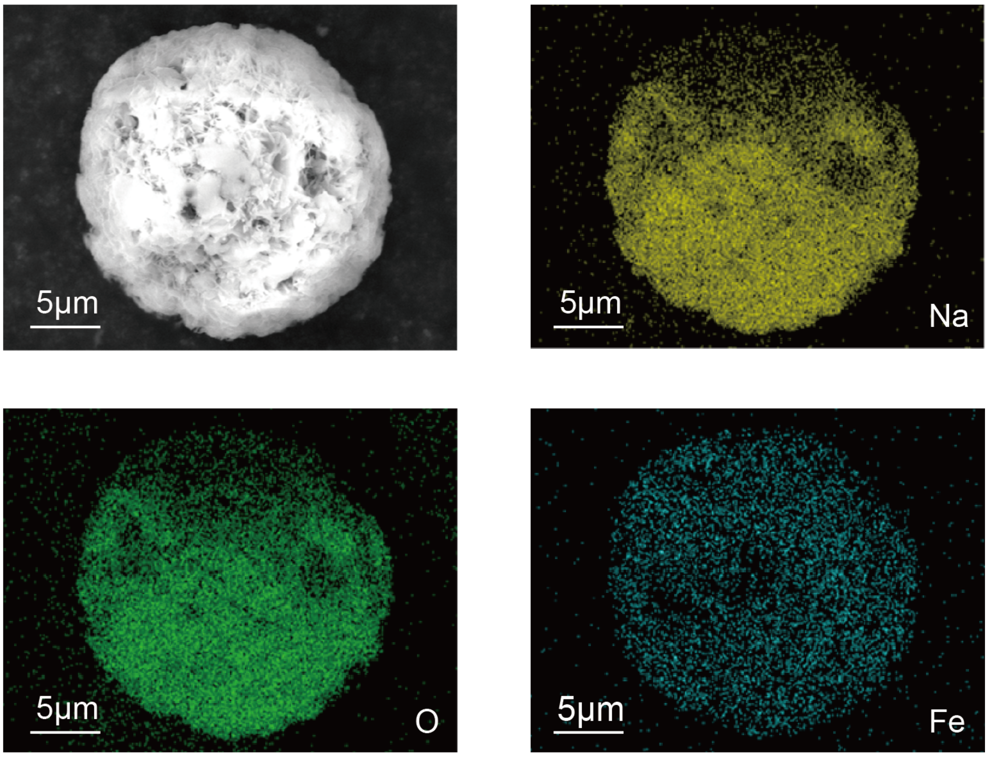

Fig. S6. SEM images and corresponding EDS elemental mappings of as-synthesized Na_4FeO_4.

Table. S7. Stoichiometry of as-synthesized Na_4FeO_4 determined by ICP.

Element	Content (mg L^{-1})	Molar ratio
**Na**	1.337	4.26
**Fe**	0.760	1

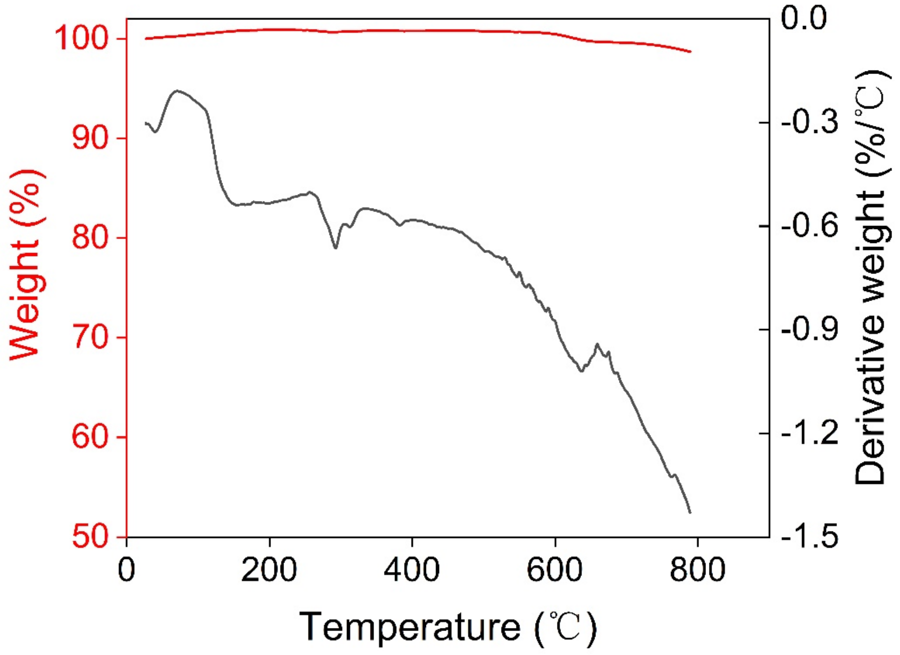

Fig. S7. TG-DTG analysis of as-synthesized Na_4FeO_4 upon heating.

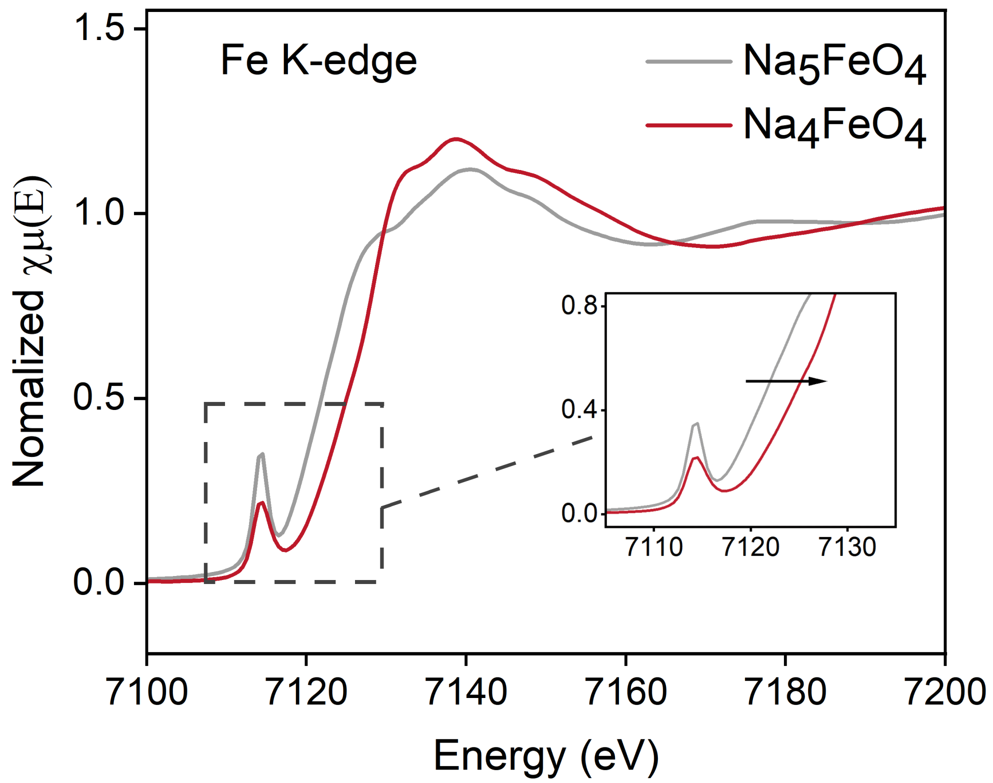

Fig. S8. Fe K-edge XANES spectra of $Na_4Fe(IV)O_4$ and $Na_5Fe(III)O_4$ (inset shows enlarged pre-edge region).

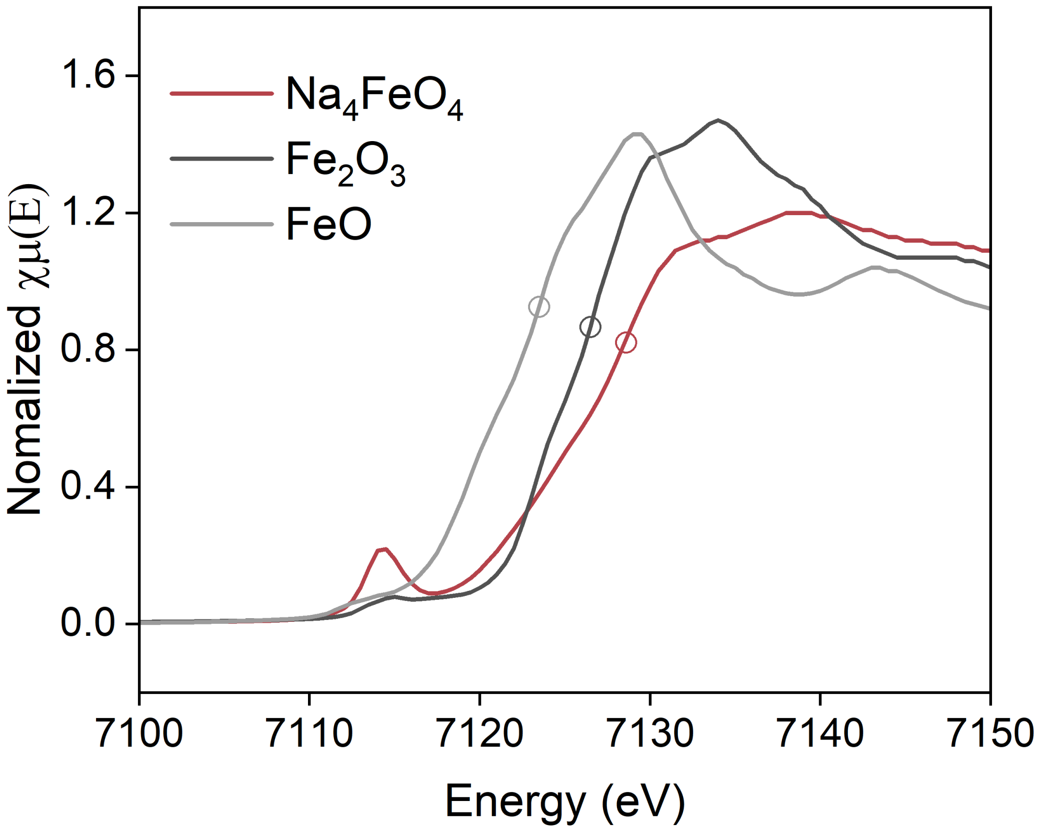

Fig. S9. Fe K-edge XANES spectra and the position of the absorption edge of FeO, Fe_2O_3 and Na_4FeO_4.

Supplementary Text 2. Calculate theoretical voltages.

The average topotactic desodiation potentials of most identified sacrificial additives (Table S1 and Fig. 1C) were derived from the energy difference between the pristine materials and their hypothetical, fully desodiated structures (complete Na removal) via equation (1)(*62*). For several ternary Na transition metal oxides of interest, detailed stepwise voltage profiles were further computed. This was achieved by enumerating and calculating intermediate structures at various desodiation levels via pymatgen (*46*).

$$V(x_1, x_2) = -\frac{E_{Na_{x1}MO_y} - E_{Na_{x2}MO_y} - (x_1 - x_2)E_{Na}}{(x_1 - x_2)F} \quad (1)$$

Na_4FeO_4: the stepwise voltage profiles via topotactic desodiation reactions were calculated by first constructing partial desodiation structures of Na_xFeO_4 for x=3.5, 3, 2, 1, 0. Various Na vacancy orderings were enumerated in a 2×1×1 supercell and pre-screened using Ewald electrostatic energy criterion via pymatgen, followed by DFT geometry optimizations. In total 15, 20, 9, 10, 1 structures for x=3.5, 3, 2, 1, 0 were calculated by DFT, respectively. The binary convex hull was shown in fig. S10, and equilibrium voltages (fig. 3B) were computed between all ground state configurations.

α-$NaFeO_2$: for the theoretical potential between α-$NaFeO_2$ and α-$Na_{0.5}FeO_2$, desodiated structures with various Na vacancy orderings were enumerated and pre-screened for α-$Na_{0.5}FeO_2$ in a 2×2×1 supercell, and 5 structures were calculated by DFT. The lowest-energy structure was used to compute the desodiation potential.

For several ternary Na transitional metal oxides including Na_4FeO_3, Na_5MnO_4 and $Na_{14}Mn_2O_9$, desodiated structures with 25% and 75% Na removal were enumerated and pre-screened using Ewald electrostatic energy. The ones with the lowest Ewald energy were selected for DFT calculations. Their resulting voltage profiles are shown in fig. S11-13, respectively.

For Na_6ZnO_4 and Na_3CuO_2, however, similar DFT calculations on the lowest-Ewald-energy structures at 25% and 75% desodiation levels produced unphysical voltage profiles. This prompted a broader structure search, leading to DFT relaxation of an additional 17 and 75 desodiated structures for Na_3CuO_2 and Na_6ZnO_4, respectively, to identify the true ground-state configurations. The corresponding convex hulls and voltage profiles are shown in fig. S14 for Na_3CuO_2 and fig. S15 for Na_6ZnO_4.

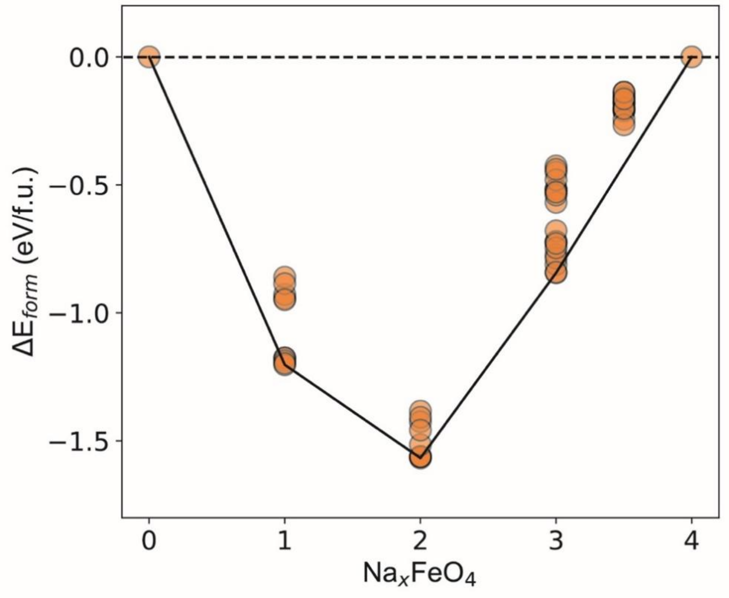

Fig. S10. The convex hull of Na_xFeO_4 (x=0.5, 1, 2, 3, 4). Ground-state compounds on the convex hull were used to calculate the theoretical voltage profiles.

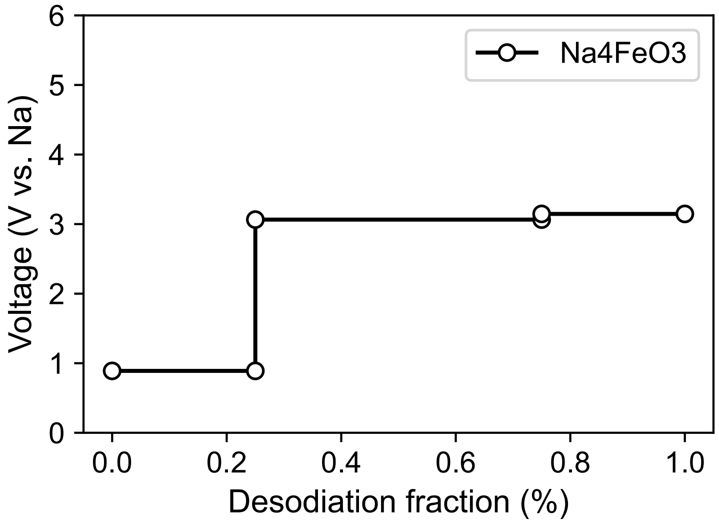

Fig. S11. Calculated voltage profile for Na_4FeO_3 based on topotactic desodiation reactions.

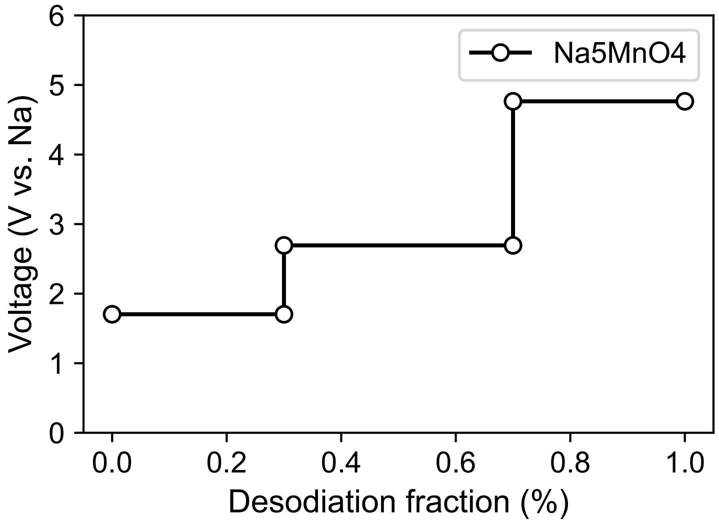

Fig. S12. Calculated voltage profile for Na_5MnO_4 based on topotactic desodiation reactions.

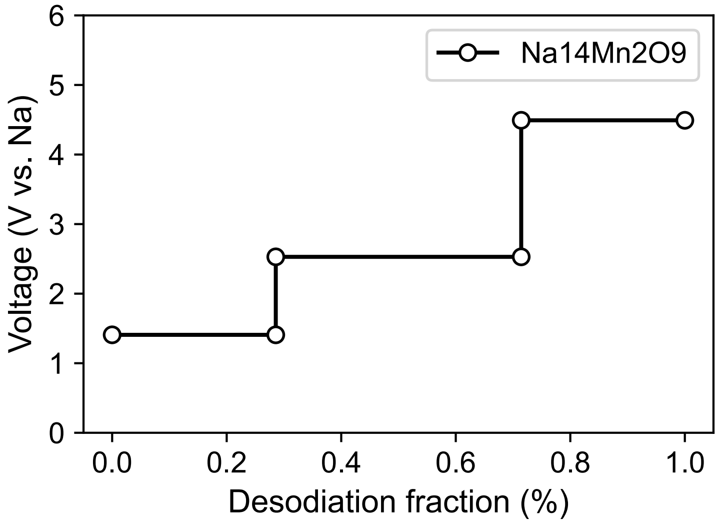

Fig. S13. Calculated voltage profile for $Na_{14}Mn_2O_9$ based on topotactic desodiation reactions.

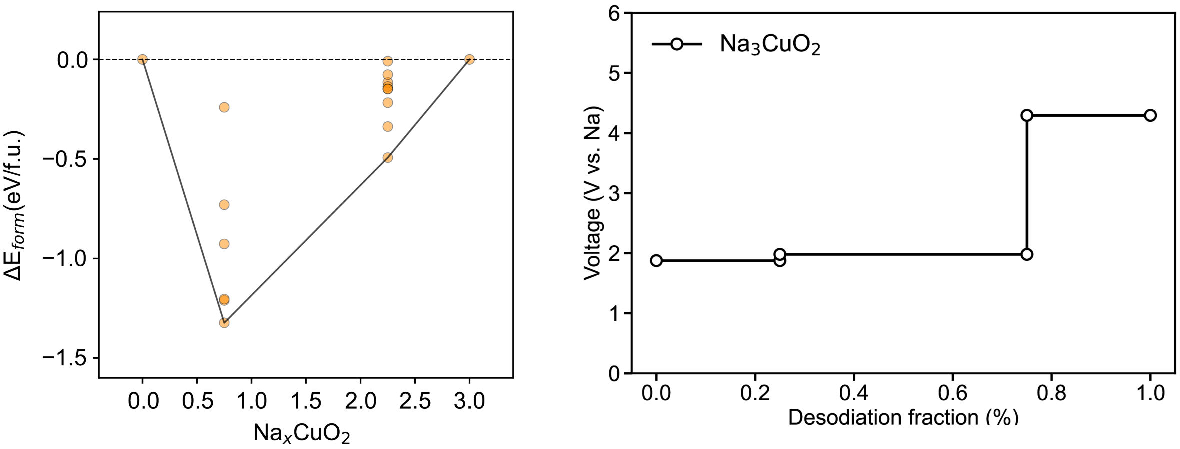

Fig. S14. The convex hull (left) of Na_xCuO_2 (x=0, 0.75, 2.25, 3). Stepwise voltage profile (right) was computed using ground-state compounds on the convex hull.

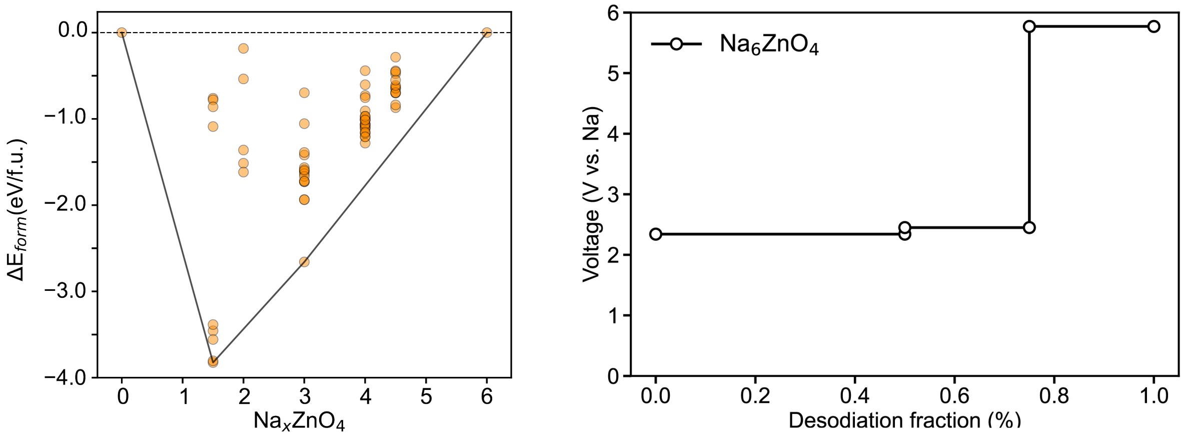

Fig. S15. The convex hull (left) of Na_xZnO_4 (x=0, 1.5, 2, 3, 4, 4.5, 6). Stepwise voltage profile (right) was computed using ground-state compounds on the convex hull.

Supplementary Text 3. Structure of β-$NaFeO_2$ from electrochemical decomposition.

The XRD patterns of the decomposition product of Na_4FeO_4 match those of β-$NaFeO_2$ (orthorhombic, *Pna21* space group), as shown in Fig. 3C. However, a noticeable deviation is observed in the relative intensity between the (200) and (112) diffraction peaks. In the standard β-$NaFeO_2$ reference, the (112) peak is more intense than the (200) peak, whereas our sample shows the opposite trend. This suggests that the $NaFeO_2$ formed upon electrochemical decomposition is not structurally identical to the standard phase.

To further investigate this, we performed Rietveld refinement on the corresponding synchrotron XRD pattern (labeled $x = 2$ in Fig. 3C), using the standard β-$NaFeO_2$ structure as the starting model. Although the presence of multiple phases, and possibly amorphous components introduced by ball milling or the electrochemical process, prevented a high-quality refinement, the analysis still provides meaningful qualitative insight into the structural differences. As shown in fig. S16A, we found that a Na-deficient β-$Na_{0.93}FeO_2$ yields a qualitatively better match for the relative peak intensities, indicating that the observed difference can be attribute to the presence of Na vacancies.

To further validate this hypothesis, we performed DFT calculations for β-$Na_{0.75}FeO_2$ (25% Na vacancies). The simulated XRD pattern show a good agreement with the experimental (200)/(112) peak intensity ratio. Furthermore, the introduced 25% Na vacancy leads to a slight splitting of the (112) peak, which is qualitatively consistent with the observed broadening of (112) peak, as compared to (200) peak. Moreover, we simulated XRD patterns of β-$Na_{0.75}FeO_2$ with different Na-vacancy configurations, which all yield similar (200)/(112) peak intensity ratios (fig. S16D).

In addition, The intensity mismatch may also arise from lattice strain or defects perturbing long-range order, as electrochemically formed phases tend to be more defective due to its nonequilibrium phase formation conditions, in contrast to the ordered lattices usually achieved via high-temperature synthesis.

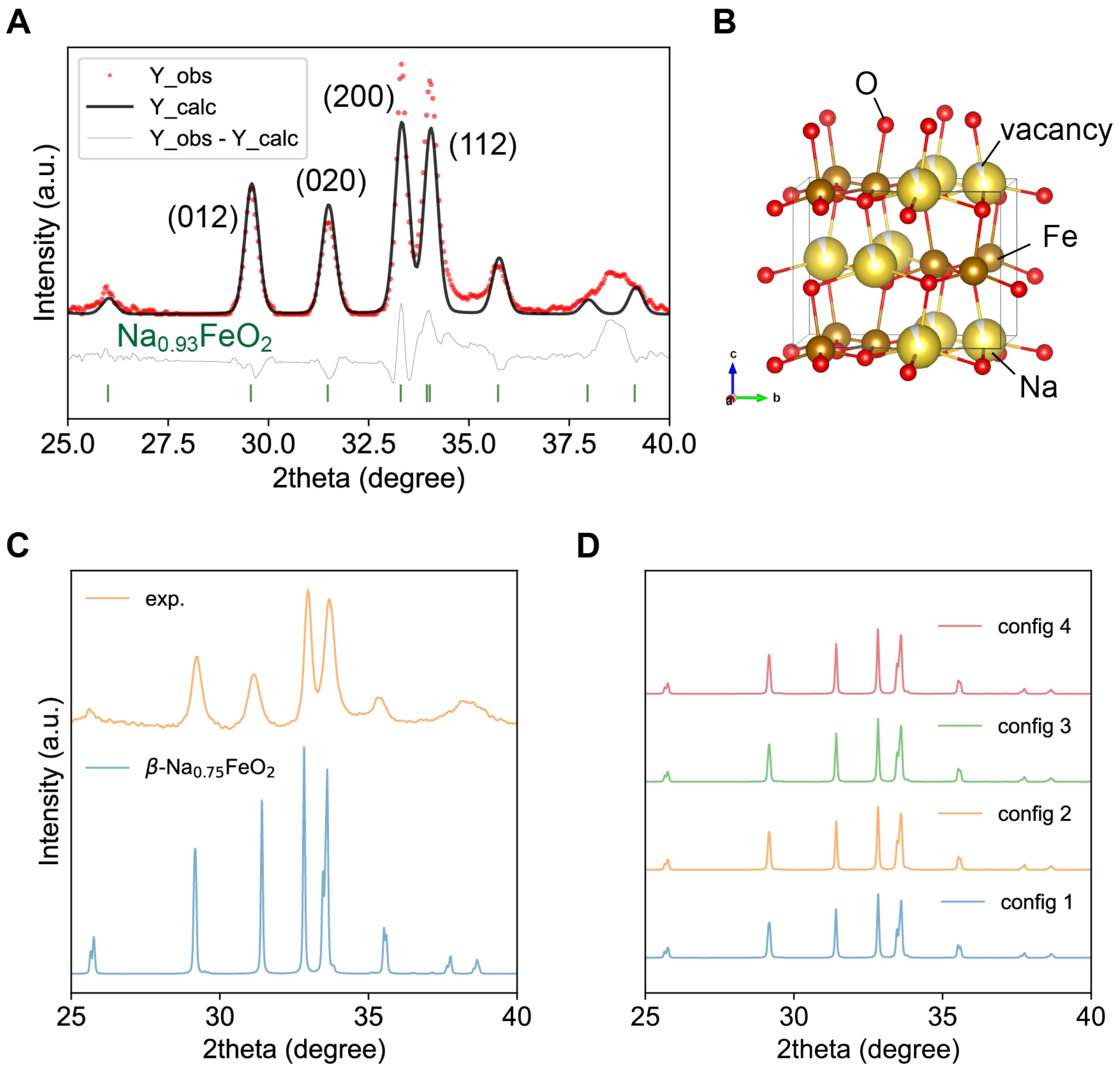

Fig. S16. Structural analysis of β-$NaFeO_2$. (A) Rietveld refinement of the electrochemically obtained β-$NaFeO_2$ using a Na-deficient β-$Na_{0.93}FeO_2$ structure. **(B)** The crystal structure of β-$Na_{0.93}FeO_2$. **(C)** Experimentally observed (top) and computationally simulated (bottom) XRD patterns. **(D)** Simulated XRD patterns for β-$Na_{0.75}FeO_2$ with various Na-vacancy configurations.

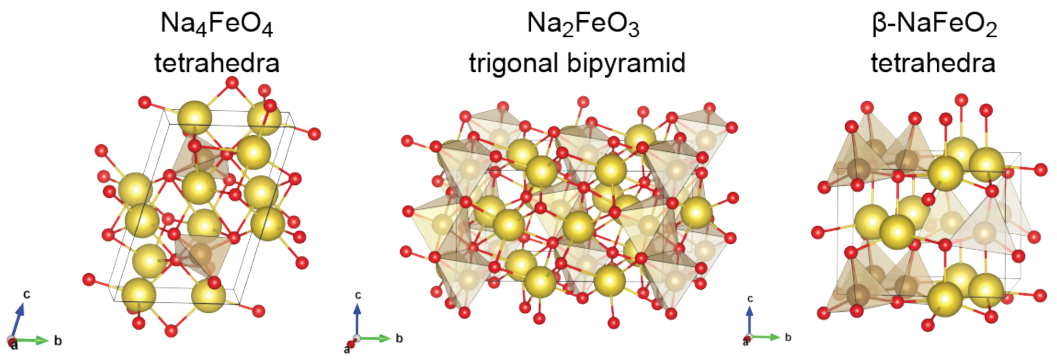

Fig. S17. Calculated decomposition products of Na_4FeO_4. Structure of Na_4FeO_4 with tetrahedrally coordinated Fe (left); structure of Na_2FeO_3 with trigonal-bipyramidal coordinated Fe (middle); structure of beta-$NaFeO_2$ with tetrahedrally coordinated Fe (right). Brown, yellow, and red spheres represent Fe, Na and O atoms, respectively.

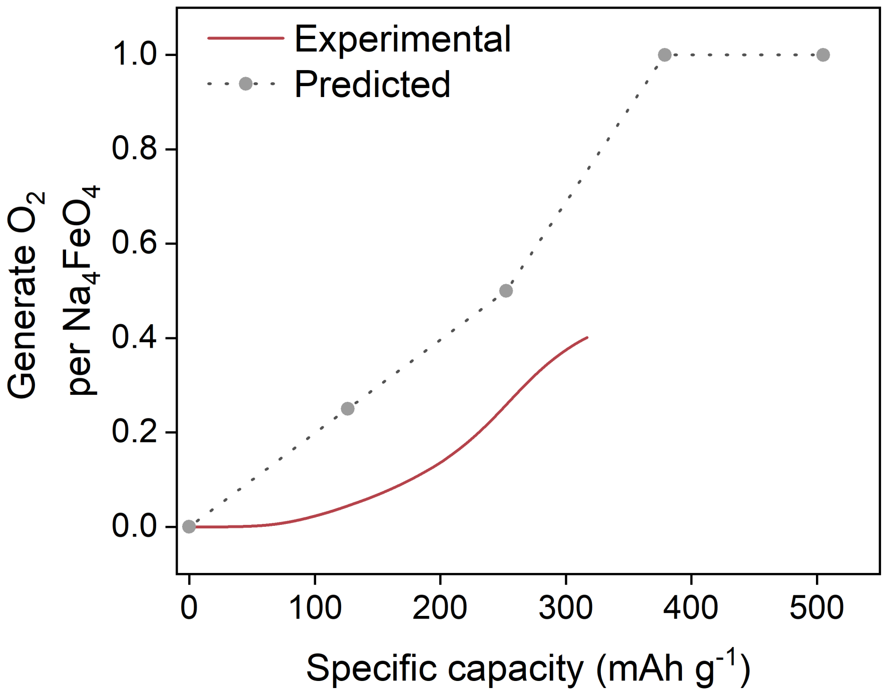

Fig. S18. Predicted and experimentally measured O_2 generation as a function of sodium extraction.

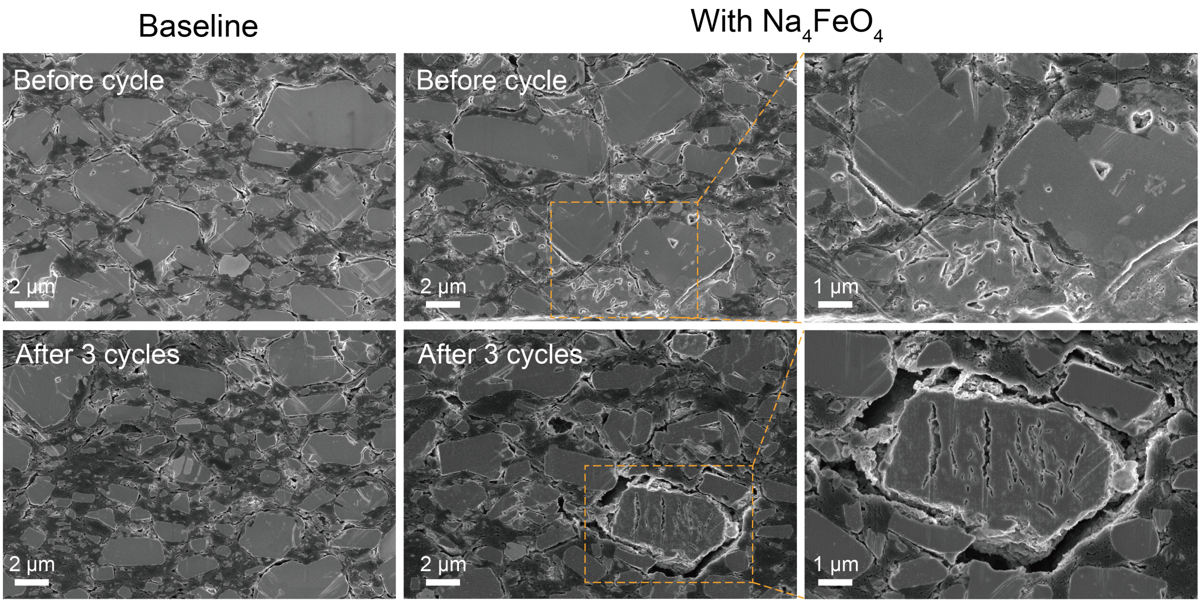

Fig. S19. Cross-sectional SEM images of NFM electrodes before and after formation cycling, for baseline full cell (left) and for full cell with the addition of NFO (right).

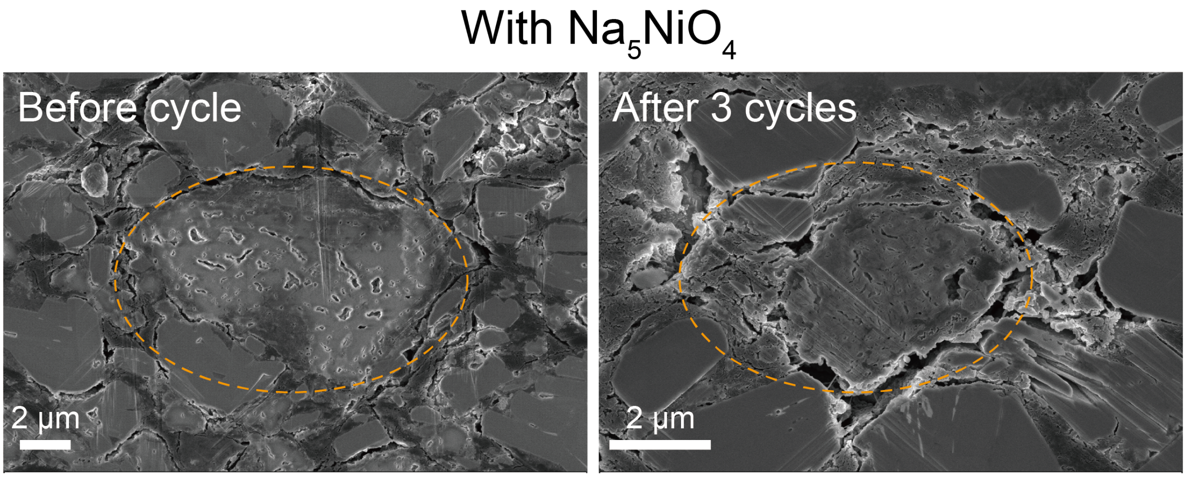

Fig. S20. Cross-sectional SEM images of NFM electrodes before and after formation cycling, for baseline full cell (left) and for full cell with the addition of Na_5NiO_4 (right).

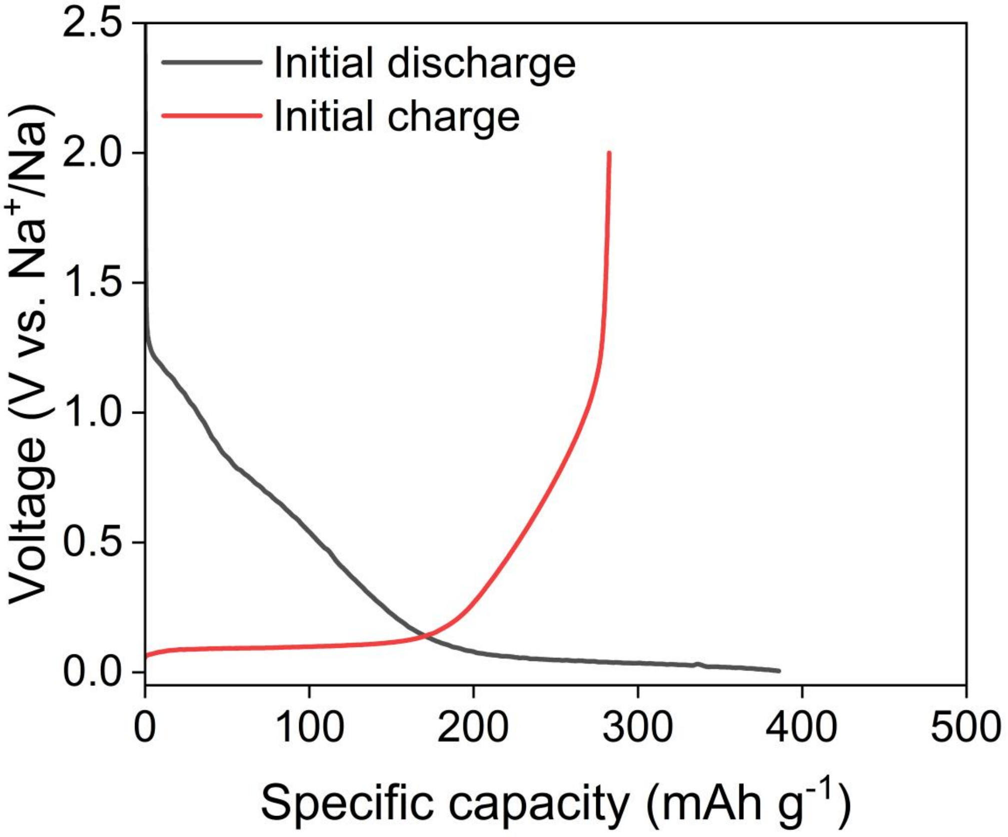

Fig. S21. Galvanostatic charge/discharge curves of HC anode at 0.1C between 0.005 and 2.0 V.

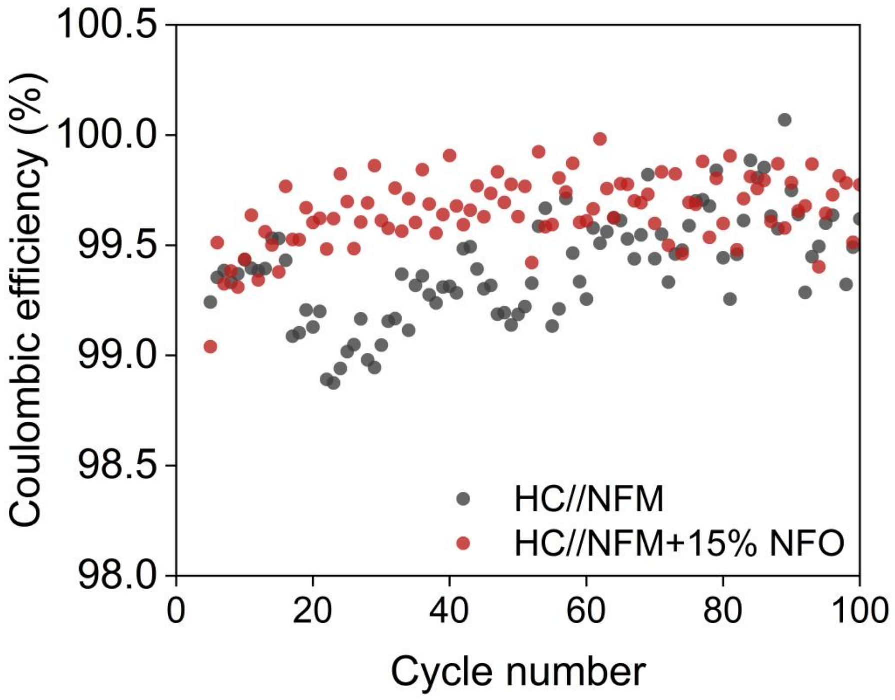

Fig. S22. Coulombic efficiencies of NFM full cells with or without NFO additives.

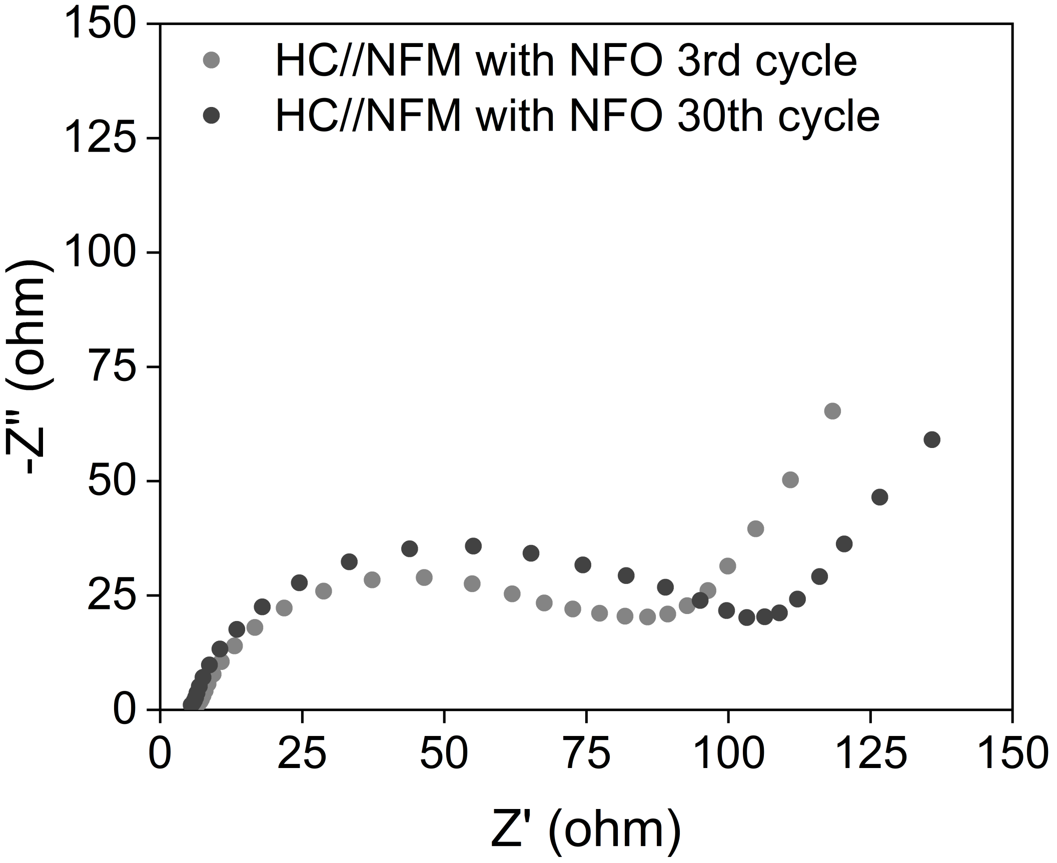

Fig. S23. EIS of full cell with NFO at the 3rd and 30th cycles.

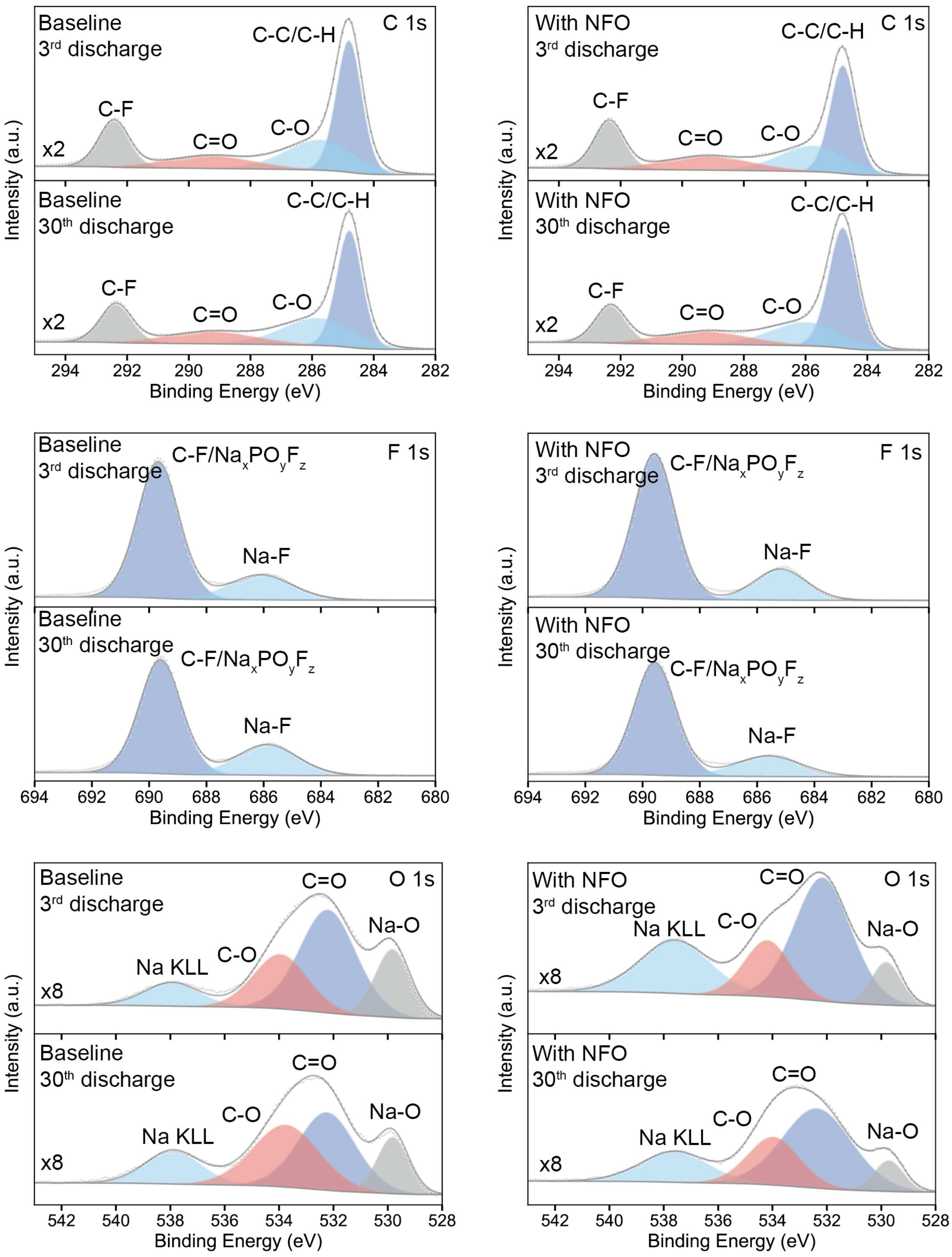

Fig. S24. CEI characterization. Cathode XPS spectra of C 1s, F 1s and O 1s peaks for baseline NFM full cells (left) and for NFM full cells with NFO (right) before and after 30 cycles.

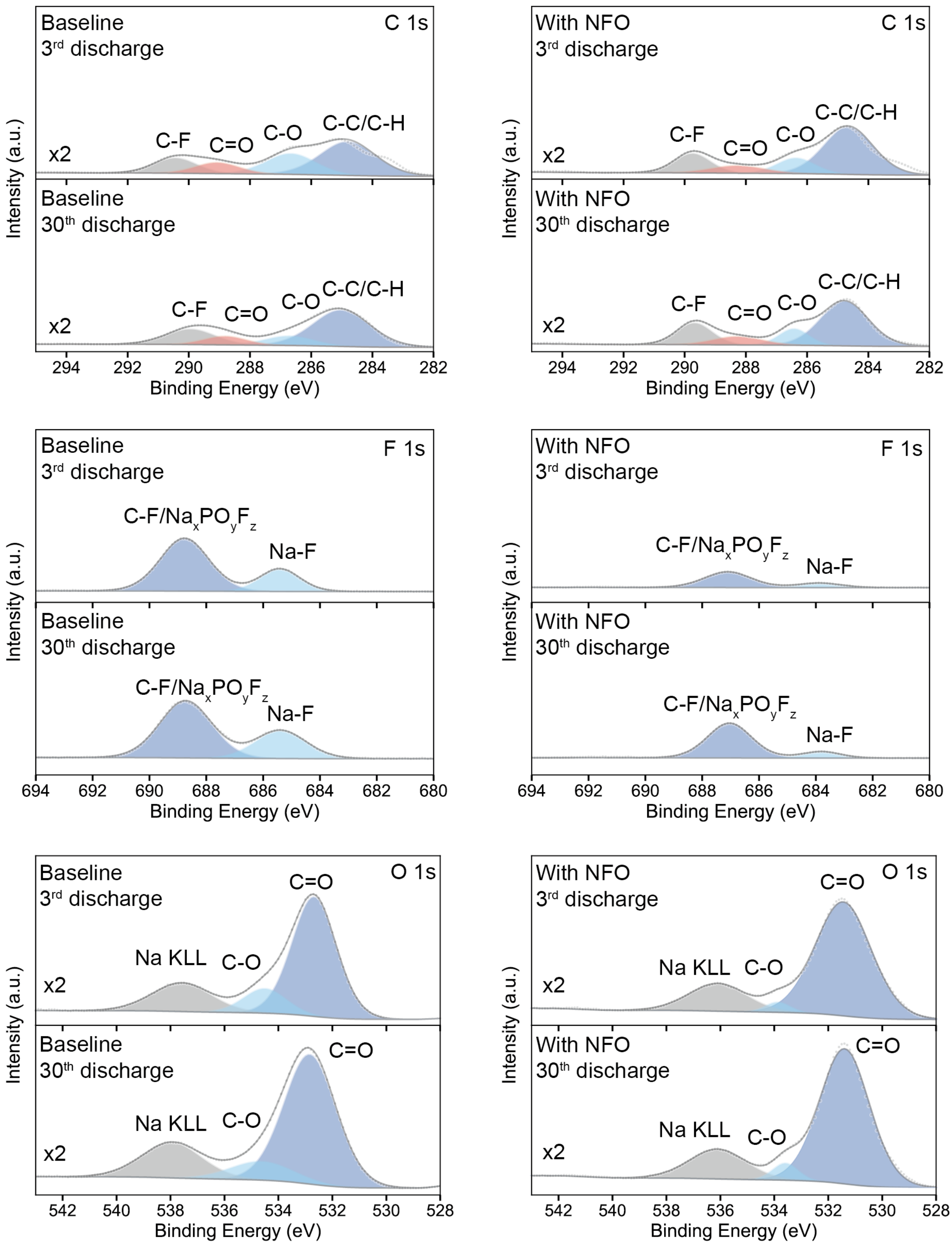

Fig. S25. SEI characterization. Cathode XPS spectra of C 1s, F 1s and O 1s peaks for baseline NFM full cells (left) and for NFM full cells with NFO (right) before and after 30 cycles.

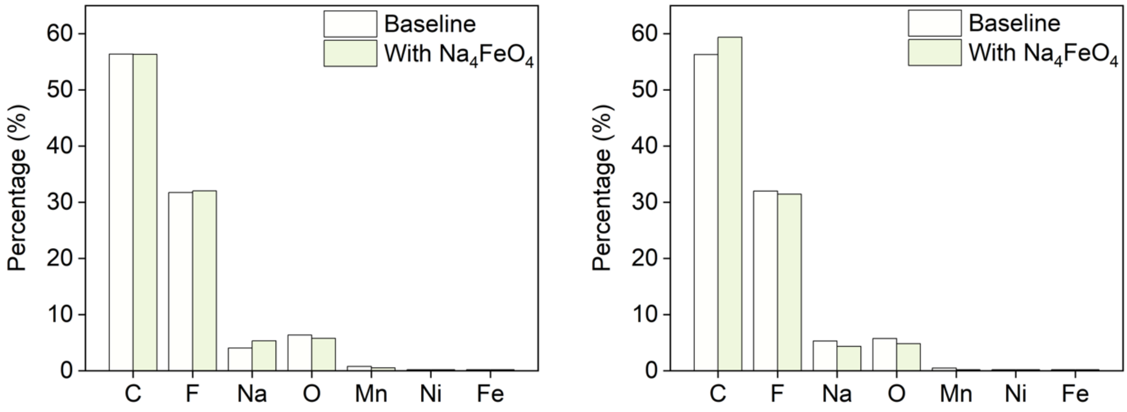

Fig. S26. CEI atomic ratio of baseline and NFO containing full cell after 3rd (left) and 30th (right) cycling.

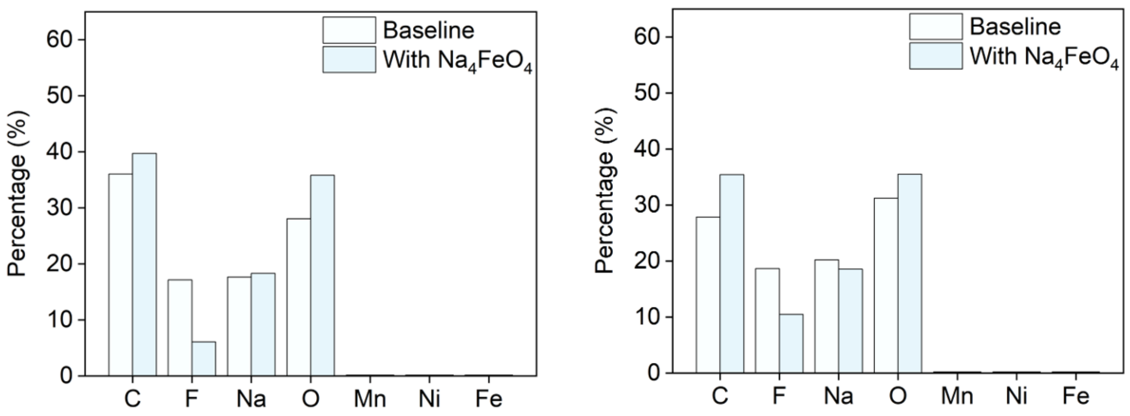

Fig. S27. SEI atomic ratio of baseline and NFO containing full cell after 3rd (left) and 30th (right) cycling.

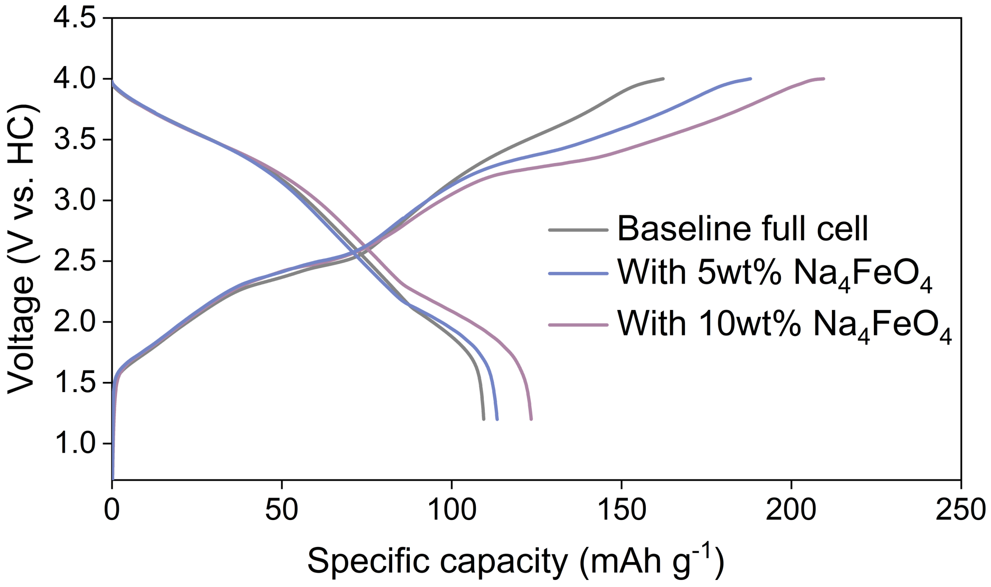

Fig. S28. The initial charge/discharge curves of the NFM full cell with 5 and 10 wt% of NFO.

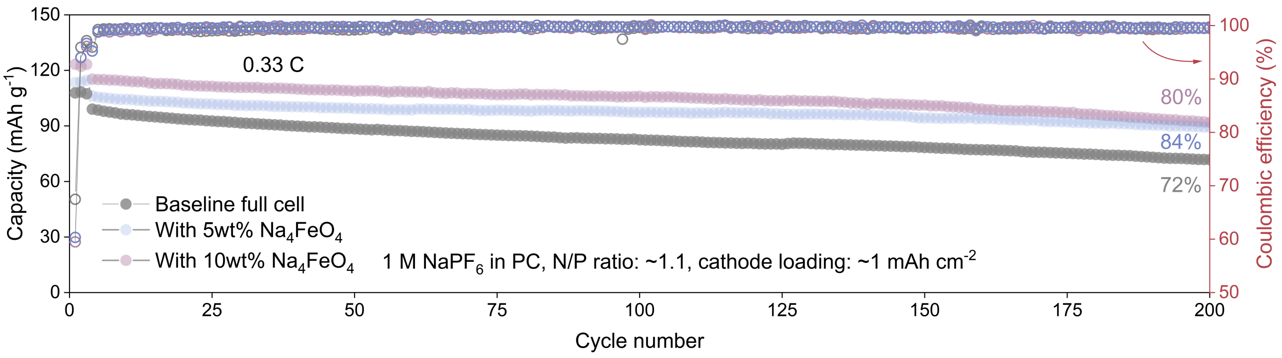

Fig. S29. Cycling performance of NFM full cells with 5 wt% and 10 wt% of NFO additives.

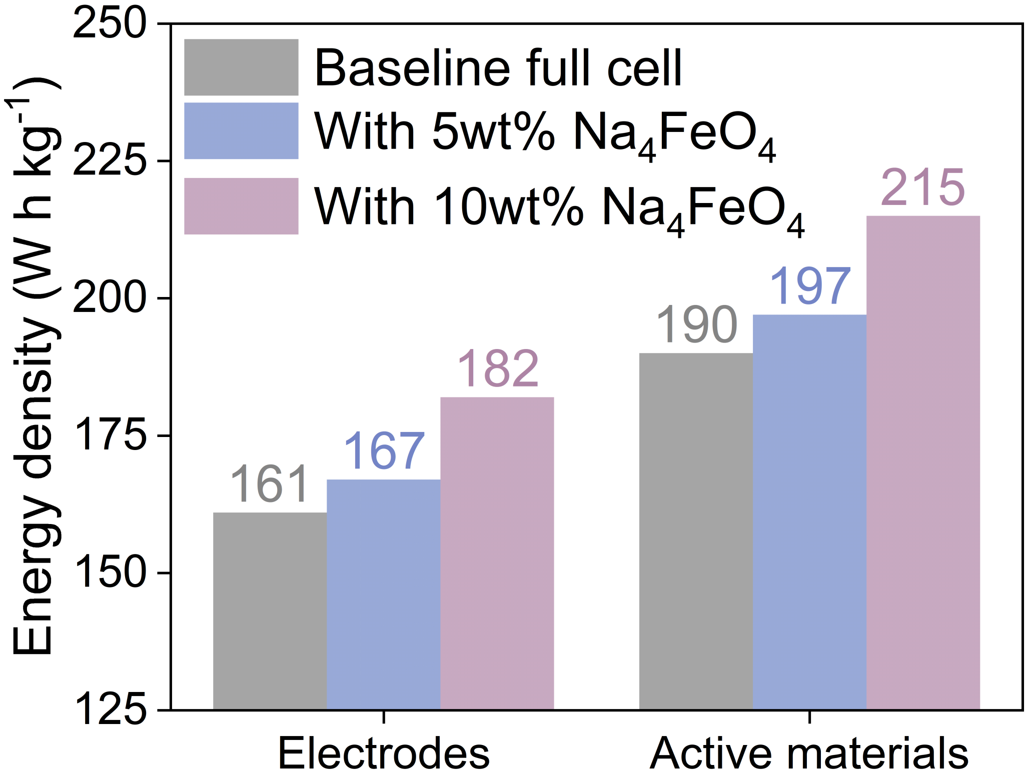

Fig. S30. Energy density comparison of NFM full cell with 5 and 10 wt% of NFO. The energy densities are normalized based on the total mass of cathode and anode electrodes (left) and the mass of cathode and anode active materials (right).

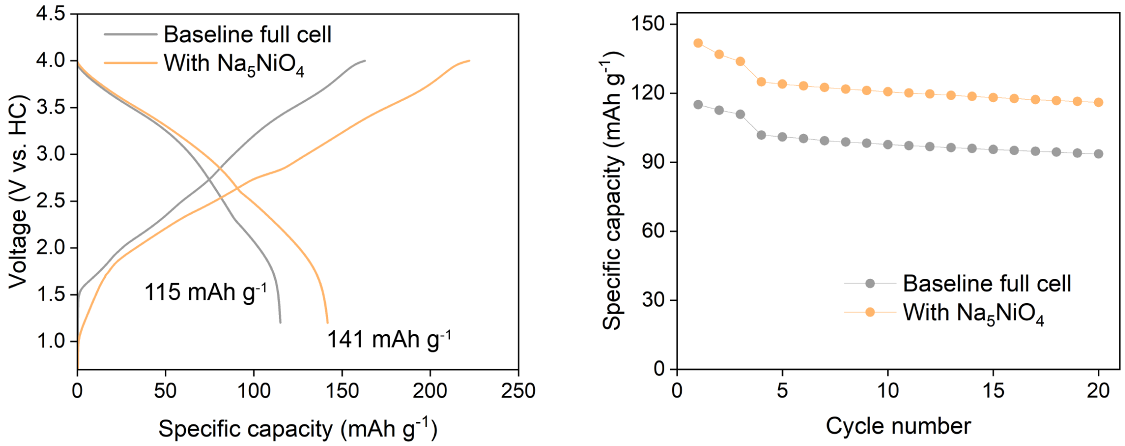

Fig. S31. The charge/discharge curves (left) and cycle stability (right) of O3-NFM full cells with 10 wt% Na_5NiO_4 additive (yellow) and without additives (grey).

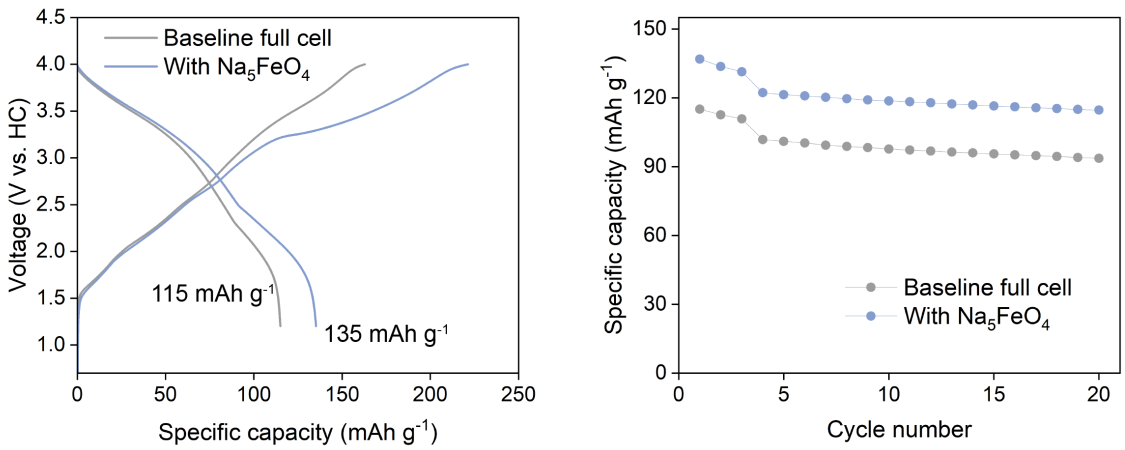

Fig. S32. The charge/discharge curves (left) and cycle stability (right) of O3-NFM full cells with 10 wt% Na_5FeO_4 additive (blue) and without additives (grey).

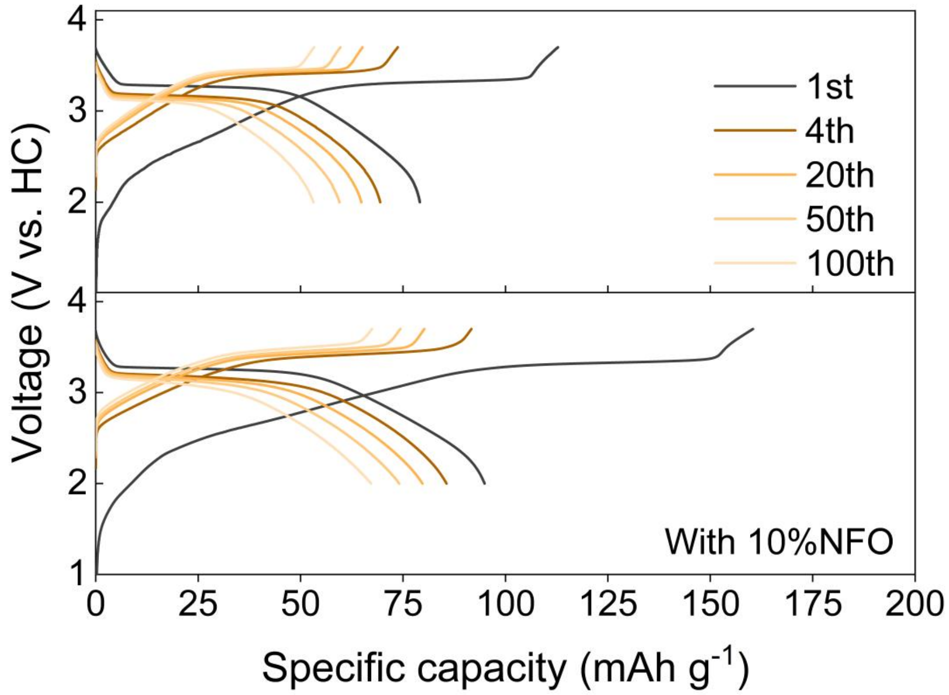

Fig. S33. The charge/discharge curves of the NVP full cell without (top) and with (bottom) 10 wt% of NFO additives.

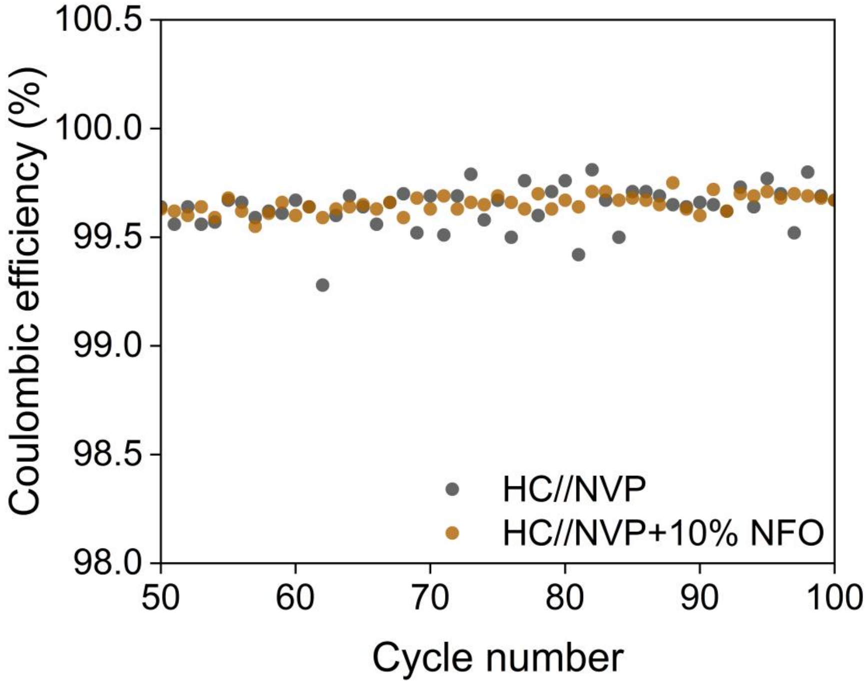

Fig. S34. Coulombic efficiencies of NVP full cells with or without NFO additives.

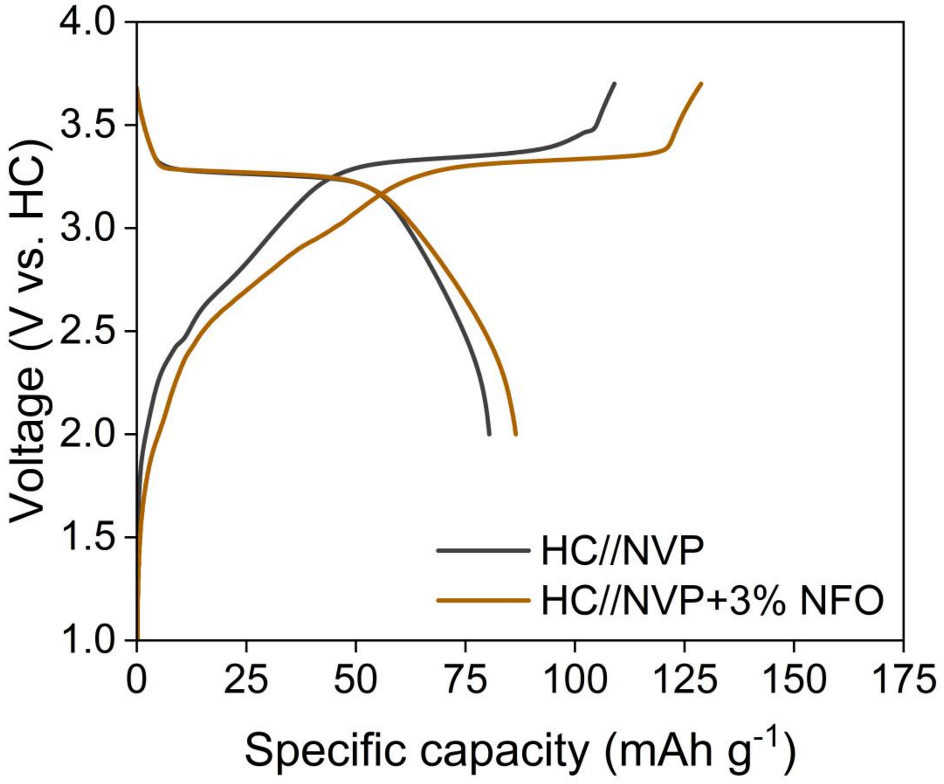

Fig. S35. The initial charge/discharge curves of NVP full cell without or with 3 wt% NFO.

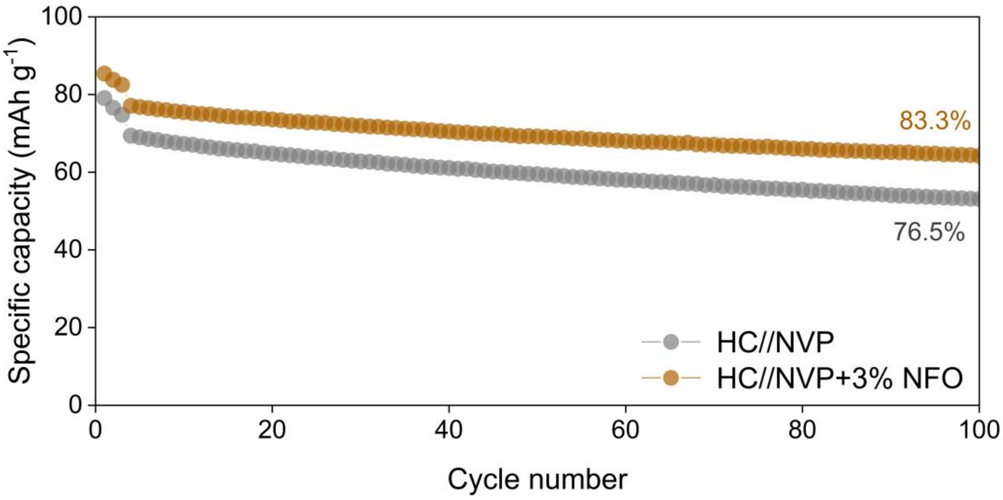

Fig. S36. Cycling performance of NVP full cell without or with 3 wt% NFO.

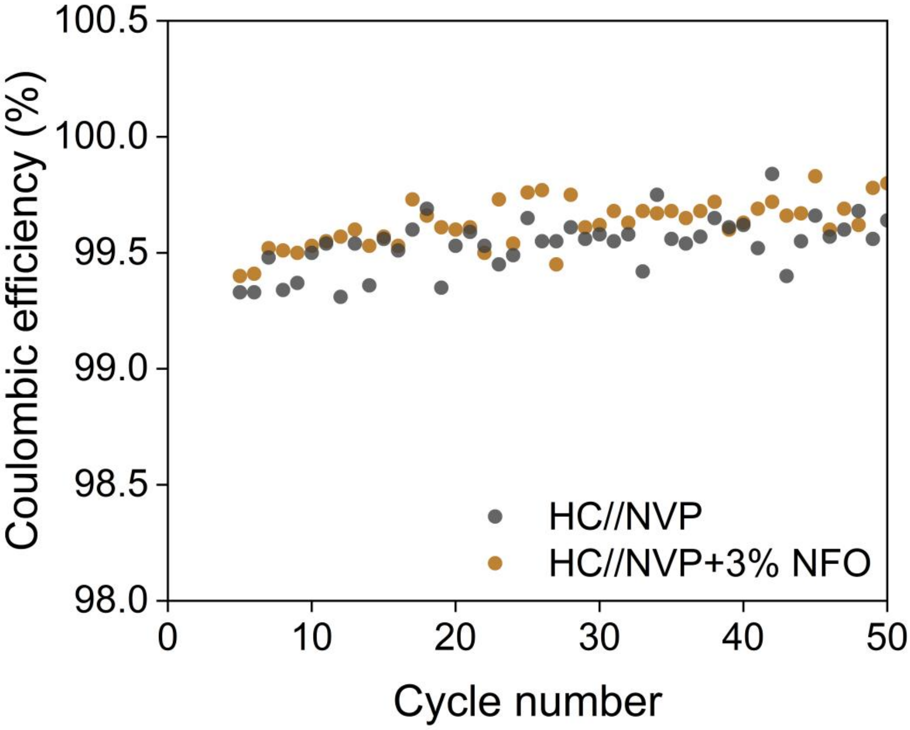

Fig. S37. Coulombic efficiencies of NVP full cells with 3 wt% or without NFO additives.

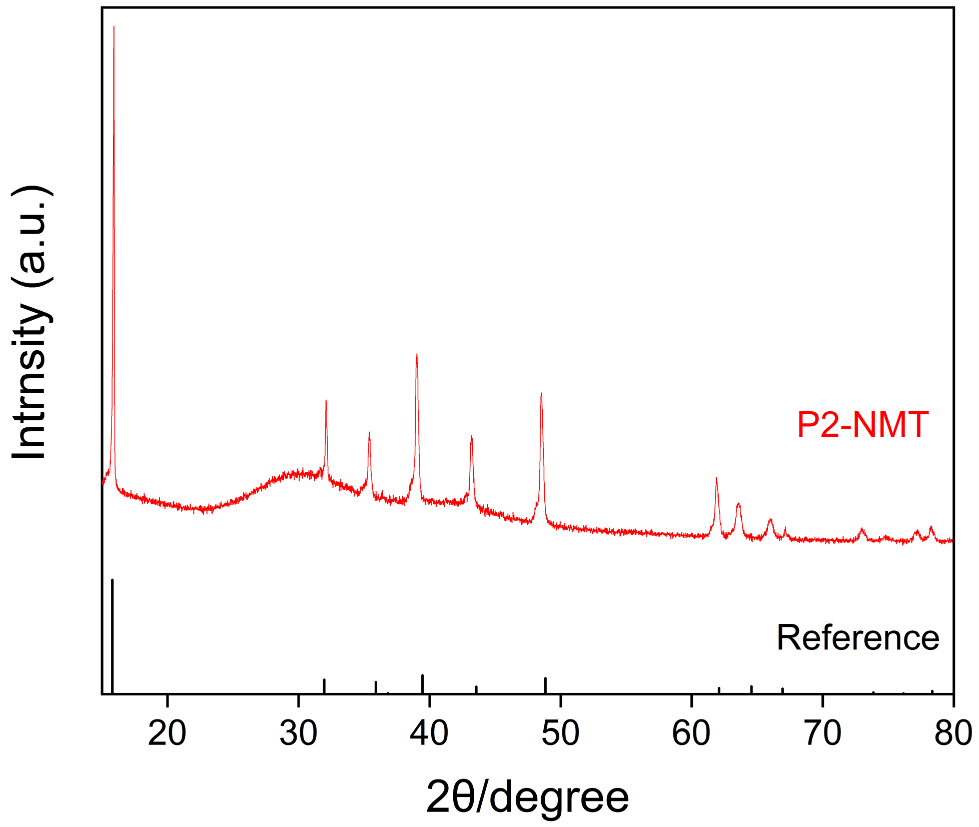

Fig. S38. XRD pattern of the as-synthesized P2-NMT cathode material.

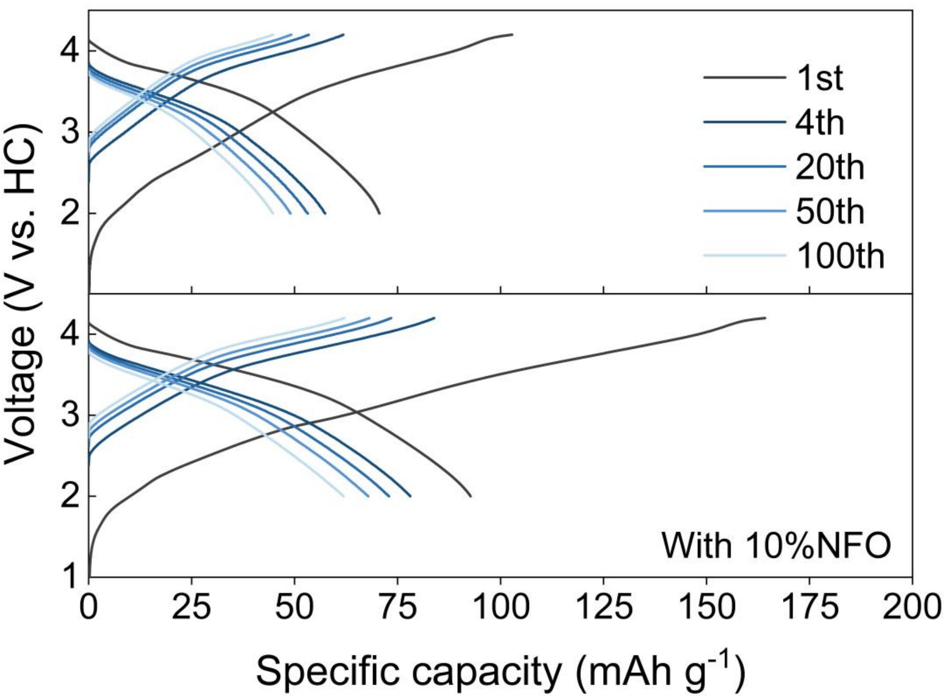

Fig. S39. The charge/discharge curves of the P2-NMT full cells without (top) and with 10 wt% (bottom) NFO.

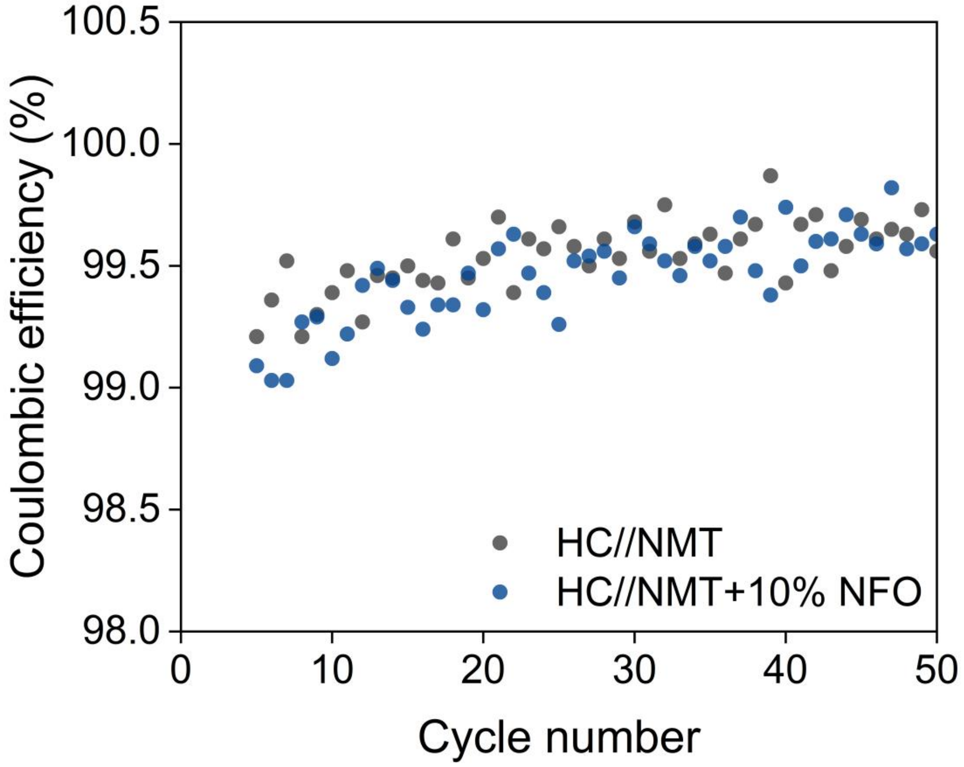

Fig. S40. Coulombic efficiencies of NMT full cells with or without NFO additives.

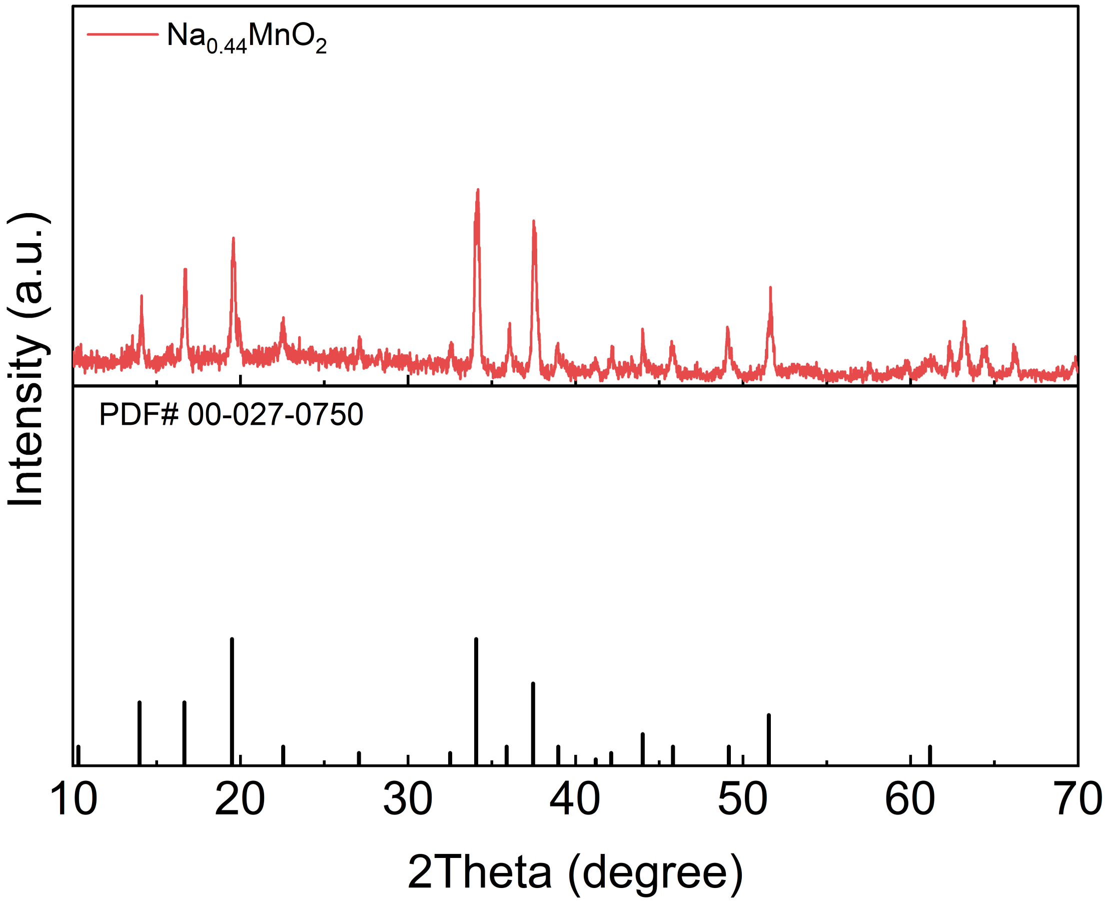

Fig. S41. XRD pattern of the as-synthesized tunnel-type $Na_{0.44}MnO_2$ cathode material.

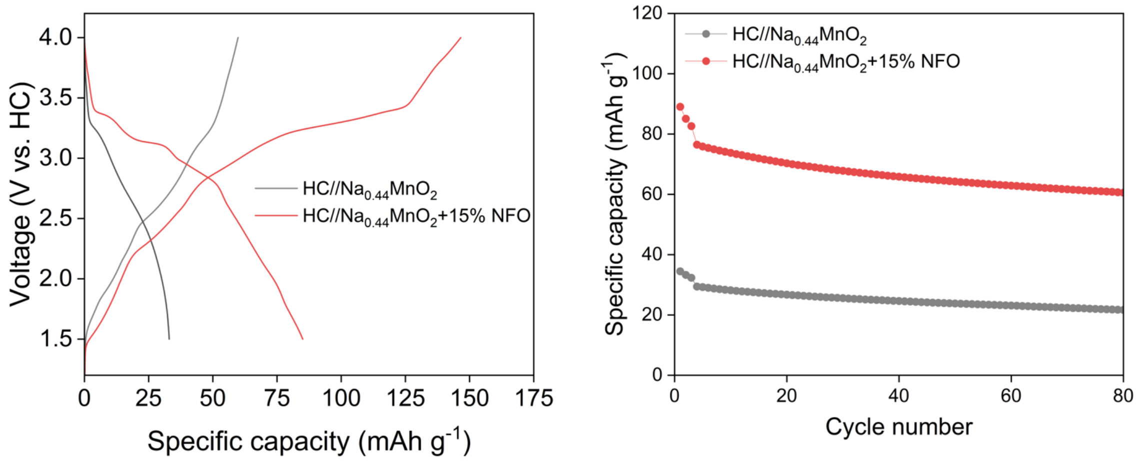

Fig. S42. The charge/discharge curves (left) and cycle stability (right) of $Na_{0.44}MnO_2$ full cells with 15 wt% Na_4FeiO_4 additive (red) and without additives (grey).

Supplementary Text 4. Energy density calculations.

Experimental: For cells without additive, the mass ratio of (active materials) : carbon : binder is set at 85:10:5 for cathode. For cells with presodiation additive, the mass ratio of (active materials) : carbon : binder is kept unchanged. Therefore, when x wt% additive is added, the mass fraction of each component in the cathode thus becomes 85:10:5:x.

The calculation of full-cell energy density is conducted using the following formula:

$$E = \frac{e}{m_a + m_c} \quad (2)$$

where E represents the gravimetric energy density; e represents the absolute energy, which can be obtained by integrating the discharge profile; m_c (m_a) represents the total mass of cathode (anode). For cells with additive, m_c includes the mass the additive unless otherwise specified.

Theoretical: For the calculations of full-cell energy density, to align with commercial battery parameters, for cells without additive, the mass ratio of (active materials) : carbon : binder is set at 94:4:2 for cathode, and 92:4:4 for anode. In this case, full-cell energy density in **Fig. 5** is calculated based on the total mass of both electrodes (cathode and anode). For cells with presodiation additive, the mass ratio of (active materials) : carbon : binder is kept unchanged. Therefore, when x wt% additive is added, the mass fraction of each component in the cathode thus becomes 94:4:2:x, and full-cell energy density is calculated based on the total mass of both electrodes and additives. A N/P ratio of 1.1 (based on the first-cycle capacity) is assumed for all calculations. Three full-cell configurations are considered:

1. For O3-NIBs, a hypothetical O3-NFM (hard carbon) is taken as cathode (anode), with an initial capacity of 160 (380) mAh g^{-1}. The coulombic efficiency of hard carbon anodes is set at 75%, while the coulombic efficiency of the NFM cathode is assumed to be 100% to simplify the calculation. The average voltage is set at 3 V. The calculated full-cell energy density as a function of the mass loading and the capacity of presodiation additives is shown in Fig. 5A.

2. For P2-NIBs, a hypothetical P2 (hard carbon) is taken as cathode (anode), with an initial capacity of 100 (380) mAh g^{-1}. The coulombic efficiency of hard carbon anodes is set at 75% as well, however, the cathode coulombic efficiency is assumed to be 150%, i.e., additional Na vacancies are present in P2 cathode. The average voltage is also set at 3 V. The calculated full-cell energy density as a function of the mass loading and the capacity of presodiation additives is shown in fig. S29.

3. For LIBs, a hypothetical LFP (graphite) is taken as cathode (anode), with an initial capacity of 160 (380) mAh g^{-1} and coulombic efficiency of 100% (92%), assuming an average voltage of 3.4 V. The calculated full-cell energy density as a function of the mass loading and the capacity of presodiation additives is shown in Fig. 5B.

Supplementary Text 5. Maximum loading of additives to prevent Na plating.

For practical full cells with a low N/P ratio, e.g., 1.1, adding too much presodiation materials may trigger potential Na plating which is detrimental to cycling stability. Therefore, in this section the maximum loading to prevent Na plating in practical full cells is calculated. Given practical full cells with a reversible N/P ratio of 1.1:

$$\frac{C_{anode}^{1st} \times m_{anode} \times \eta_{anode}}{C_{cath}^{1st} \times m_{cath} \times \eta_{cath}} = 1.1$$

$$C_{anode}^{1st} \times m_{anode} = 1.1 \frac{\eta_{cath}}{\eta_{anode}} \times C_{cath}^{1st} \times m_{cath} \quad (3)$$

C_{anode}^{1st} (C_{cath}^{1st}), m_{anode}, (m_{cath}) and η_{anode}, (η_{cath}) refer to the first-cycle specific capacity (mAh g^{-1}), mass (g) and initial coulombic efficiency of anode and cathode, respectively. To strictly prevent Na plating, the first-charge N/P ratio must be greater than 1:

$$\frac{C_{anode}^{1st} \times m_{anode}}{C_{cath}^{1st} \times m_{cath} + C_{NFO} \times m_{NFO}} \geq 1$$

$$m_{NFO} \leq \frac{C_{anode}^{1st} \times m_{anode} - C_{cath}^{1st} \times m_{cath}}{C_{NFO}} \quad (4)$$

where C_{NFO} and m_{NFO} refer to the specific presodiation capacity (mAh g^{-1}) and mass (g) of additive. Combining equation (3) and (4):

$$m_{NFO} \leq \frac{(1.1 \frac{\eta_{cath}}{\eta_{anode}} - 1) \times C_{cath}^{1st} \times m_{cath}}{C_{NFO}}$$

$$\frac{m_{NFO}}{m_{cath}} \leq (1.1 \frac{\eta_{cath}}{\eta_{anode}} - 1) \frac{C_{cath}^{1st}}{C_{NFO}} \quad (5)$$

For a practical O3-NFM||hard carbon full cell, we assume O3-NFM exhibits an initial capacity of 160 mAh g^{-1}, with an initial coulombic efficiency of 95%. The coulombic efficiency for hard carbon anode is set to be 75%. With NFO delivers a presodiation capacity of 450 mAh g^{-1}, the maximum $\frac{m_{NFO}}{m_{cath}}$ is calculated to be ~13.99 wt%.

For comparison, in practical LFP||graphite full cell, we assume LFP exhibits an initial capacity of 160 mAh g^{-1} and initial coulombic efficiency of 95%. The coulombic efficiency for graphite anode is set to be 92%. With Li_5FeO_4 delivers a prelithiation capacity of 650 mAh g^{-1}, the maximum $\frac{m_{NFO}}{m_{cath}}$ is calculated to be ~3.34 wt%.

The maximum loading of presodiation (prelithiation) additives with different compensation capacity is plotted in Fig. S43.

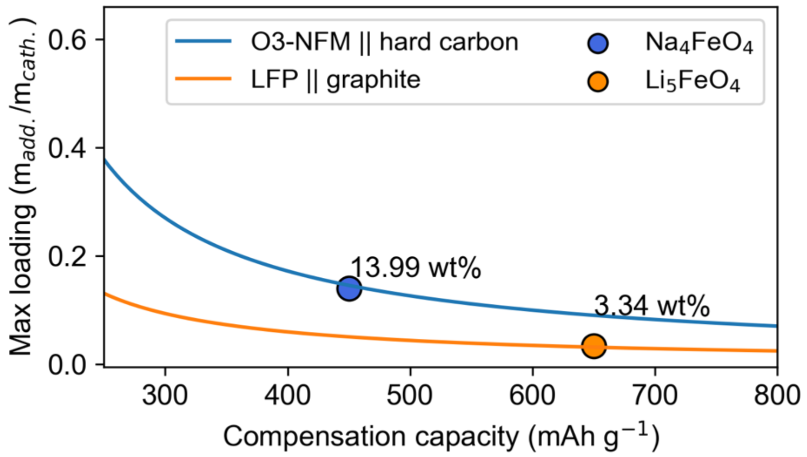

Fig. S43. The maximum loading of presodiation (prelithiation) additives as a function of presodiation (prelithiation) capacity for practical NIB (LIB) with a N/P ratio of 1.1.

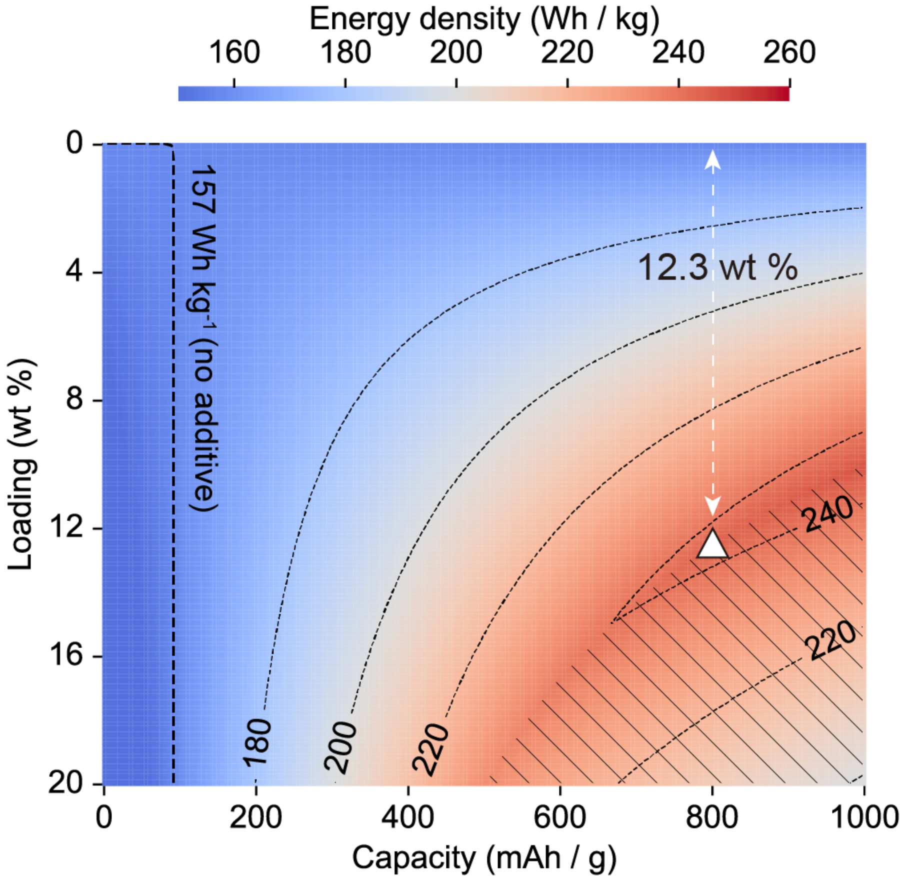

Fig. S44. Contour map depicting the energy density as a function of the mass loading and the capacity of presodiation additives for P2|HC Na-ion full cells.

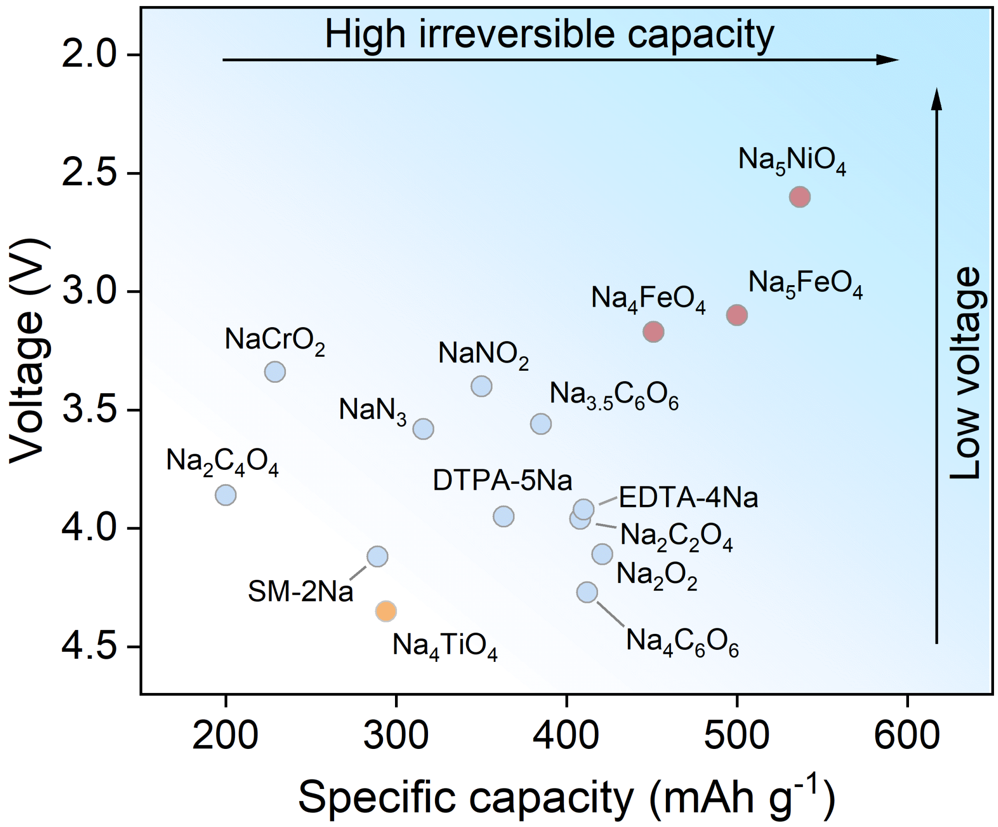

Fig. S45. Comparison of the specific capacity and average decomposition voltage of the Na_4FeO_4, Na_5FeO_4, Na_5NiO_4 and Na_4TiO_4 additive with those reported in literature (the relevant references are listed in Table S8).

Table S8. Comparison of the performances of this work and the reported sacrificial additives.

Additive	Decomposition capacity (mAh g^{-1})	Average voltage (V)	Voltage range (V)	Ref.
Na_4FeO_4	451	3.17	2-4.5	This work
Na_5FeO_4	500	3.10	2-4.5	This work
Na_5NiO_4	537	2.60	2-4.5	This work
Na_4TiO_4	294	4.35	2-4.8	This work
NaAc	323	3.95	2-4.3	(*63*)
$Na_2C_2O_4$	400	4.11	2-4.5	(*24*)
$NaNO_2$	350	3.3	2.5-4.3	(*52*)
Na_2NiO_2	290	2.2	1.15-3.8	(*22*)
Na_2O_2	421.5	4.1	2.8-4.4	(*21*)
NaN_3	315	3.5	2.6-4.3	(*64*)
$Na_{3.5}C_6O_6$	385	3.5	2.5-4.5	(*65*)
$Na_4C_6O_6$	412	3.56	2-3.4	(*66*)
DTPA-5Na	363	3.95	2.5-4.3	(*27*)
SM-2Na	289	4.12	2-4.3	(*28*)
$NaCrO_2$	229	3.34	2-4.2	(*19*)
EDTA-4Na	410	3.92	2-4.5	(*26*)
$Na_2C_4O_4$	200	3.86	2.2-4.2	(*25*)

Table S9. Theoretical and measured capacity of the synthesized presodiation additives.

**Compound**	**Theoretical capacity (mAh g^{-1})**	**Measured capacity at 4.5 V (mAh g^{-1})**	**Decomposition ratio**
Na_4FeO_4	506	451	89.1%
Na_4TiO_4	508	251	49.4%
Na_5NiO_4	568	537	94.5%
Na_5FeO_4	570	479	84.0%

Data S1. (separate file)

The raw data needed to evaluate and reproduce the results in the paper.

REFERENCES

1. C. Zhao, Q. Wang, Z. Yao, J. Wang, B. Sanchez-Lengeling, F. Ding, X. Qi, Y. Lu, X. Bai, B. Li, H. Li, A. Aspuru-Guzik, X. Huang, C. Delmas, M. Wagemaker, L. Chen, Y.-S. Hu, Rational design of layered oxide materials for sodium-ion batteries. *Science* **370**, 708–711 (2020).

2. Y.-J. Guo, R.-X. Jin, M. Fan, W.-P. Wang, S. Xin, L.-J. Wan, Y.-G. Guo, Sodium layered oxide cathodes: Properties, practicality and prospects. *Chem. Soc. Rev.* **53**, 7828–7874 (2024).

3. R. Usiskin, Y. Lu, J. Popovic, M. Law, P. Balaya, Y.-S. Hu, J. Maier, Fundamentals, status and promise of sodium-based batteries. *Nat. Rev. Mater.* **6**, 1020–1035 (2021).

4. S. Chu, S. Guo, H. Zhou, Advanced cobalt-free cathode materials for sodium-ion batteries. *Chem. Soc. Rev.* **50**, 13189–13235 (2021).

5. L. Xiao, H. Lu, Y. Fang, M. L. Sushko, Y. Cao, X. Ai, H. Yang, J. Liu, Low-defect and low-porosity hard carbon with high coulombic efficiency and high capacity for practical sodium ion battery anode. *Adv. Energy Mater.* **8**, 1703238 (2018).

6. Z. Tang, R. Zhang, H. Wang, S. Zhou, Z. Pan, Y. Huang, D. Sun, Y. Tang, X. Ji, K. Amine, M. Shao, Revealing the closed pore formation of waste wood-derived hard carbon for advanced sodium-ion battery. *Nat. Commun.* **14**, 6024 (2023).

7. T. Feng, Y. Xu, Z. Zhang, X. Du, X. Sun, L. Xiong, R. Rodriguez, R. Holze, Low-cost Al_2O_3 coating layer as a preformed SEI on natural graphite powder to improve coulombic efficiency and high-rate cycling stability of lithium-ion batteries. *ACS Appl. Mater. Interfaces* **8**, 6512–6519 (2016).

8. X. Zhao, Y. Ding, Q. Xu, X. Yu, Y. Liu, H. Shen, Low-temperature growth of hard carbon with graphite crystal for sodium-ion storage with high initial coulombic efficiency: A general method. *Adv. Energy Mater.* **9**, 1803648 (2019).

9. Y. Yang, C. Wu, X.-X. He, J. Zhao, Z. Yang, L. Li, X. Wu, L. Li, S.-L. Chou, Boosting the development of hard carbon for sodium-ion batteries: Strategies to optimize the initial coulombic efficiency. *Adv. Funct. Mater.* **34**, 2302277 (2024).

10. M. Fei, L. Qi, S. Han, Y. Li, H. Xi, Z. Lin, J. Wang, C. Ducati, M. Chhowalla, R. V. Kumar, Y. Jin, J. Zhu, Preformation of insoluble solid-electrolyte interphase for highly reversible na-ion batteries. *Angew. Chem. Int. Ed.* **63**, e202409719 (2024).

11. Y. Tian, G. Zeng, A. Rutt, T. Shi, H. Kim, J. Wang, J. Koettgen, Y. Sun, B. Ouyang, T. Chen, Z. Lun, Z. Rong, K. Persson, G. Ceder, Promises and challenges of next-generation "beyond Li-ion" batteries for electric vehicles and grid decarbonization. *Chem. Rev.* **121**, 1623–1669 (2021).

12. N. Zhang, X. Dong, Q. Yan, J. Wang, F. Jin, J. Liu, D. Wang, H. Liu, B. Wang, S. Dou, High-entropy doping NASICON cathode breaks the kinetic barriers and suppresses voltage hysteresis for sodium ion batteries. *Energy Storage Mater.* **72**, 103734 (2024).

13. S. Lin, H. Zhang, C. Shu, W. Hua, X. Wang, Y. Zhao, J. Luo, Z. Tang, Y. Wu, W. Tang, Research progress and perspectives on pre-sodiation strategies for sodium-ion batteries. *Adv. Funct. Mater.* **34**, 2409628 (2024).

14. P. Kulkarni, H. Jung, D. Ghosh, M. Jalalah, M. Alsaiari, F. A. Harraz, R. G. Balakrishna, A comprehensive review of pre-lithiation/sodiation additives for Li-ion and Na-ion batteries. *J. Energy Chem.* **76**, 479–494 (2023).

15. H. He, D. Sun, Y. Tang, H. Wang, M. Shao, Understanding and improving the initial Coulombic efficiency of high-capacity anode materials for practical sodium ion batteries. *Energy Storage Mater.* **23**, 233–251 (2019).

16. J. Hu, L. Xu, X. Li, Q. Liang, C. Ding, Y. Li, Y. Liu, Y. Gao, Pre-sodiation strategies for constructing high-performance sodium-ion batteries. *J. Mater. Chem. A* **13**, 3206–3235 (2025).

17. Y. Guo, X. Li, Z. Wang, H. Guo, J. Wang, Bifunctional Li_6CoO_4 serving as prelithiation reagent and pseudocapacitive electrode for lithium ion capacitors. *J. Energy Chem.* **47**, 38–45 (2020).

18. B. Zhu, N. Wen, J. Wang, Q. Wang, J. Zheng, Z. Zhang, Defect engineering of air-stable Li_5FeO_4 towards an ultra-high capacity cathode prelithiation additive. *Chem. Sci.* **15**, 12879–12888 (2024).

19. B. Shen, R. Zhan, C. Dai, Y. Li, L. Hu, Y. Niu, J. Jiang, Q. Wang, M. Xu, Manipulating irreversible phase transition of $NaCrO_2$ towards an effective sodium compensation additive for superior sodium-ion full cells. *J. Colloid Interface Sci.* **553**, 524–529 (2019).

20. M. Sathiya, J. Thomas, D. Batuk, V. Pimenta, R. Gopalan, J.-M. Tarascon, Dual stabilization and sacrificial effect of Na_2CO_3 for increasing capacities of na-ion cells based on P2-Na_xMO_2 electrodes. *Chem. Mater.* **29**, 5948–5956 (2017).

21. Y.-J. Guo, Y.-B. Niu, Z. Wei, S.-Y. Zhang, Q. Meng, H. Li, Y.-X. Yin, Y.-G. Guo, Insights on electrochemical behaviors of sodium peroxide as a sacrificial cathode additive for boosting energy density of Na-ion battery. *ACS Appl. Mater. Interfaces* **13**, 2772–2778 (2021).

22. K. Park, B.-C. Yu, J. B. Goodenough, Electrochemical and chemical properties of Na_2NiO_2 as a cathode additive for a rechargeable sodium battery. *Chem. Mater.* **27**, 6682–6688 (2015).

23. G. Singh, B. Acebedo, M. Casas Cabanas, D. Shanmukaraj, M. Armand, T. Rojo, An approach to overcome first cycle irreversible capacity in P2-$Na_{2/3}Fe_{1/2}Mn_{1/2}O_2$. *Electrochem. Commun.* **37**, 61–63 (2013).

24. Y. B. Niu, Y. J. Guo, Y. X. Yin, S. Y. Zhang, T. Wang, P. Wang, S. Xin, Y. G. Guo, High-efficiency cathode sodium compensation for sodium-ion batteries. *Adv. Mater.* **32**, e2001419 (2020).

25. J. Martínez De Ilarduya, L. Otaegui, M. Galcerán, L. Acebo, D. Shanmukaraj, T. Rojo, M. Armand, Towards high energy density, low cost and safe Na-ion full-cell using P2–$Na_{0.67}[Fe_{0.5}Mn_{0.5}]O_2$ and $Na_2C_4O_4$ sacrificial salt. *Electrochim. Acta* **321**, 134693 (2019).

26. J. H. Jo, J. U. Choi, Y. J. Park, J. Zhu, H. Yashiro, S.-T. Myung, New insight into ethylenediaminetetraacetic acid tetrasodium salt as a sacrificing sodium ion source for sodium-deficient cathode materials for full cells. *ACS Appl. Mater. Interfaces* **11**, 5957–5965 (2019).

27. J. H. Jo, J. U. Choi, Y. J. Park, J. K. Ko, H. Yashiro, S.-T. Myung, A new pre-sodiation additive for sodium-ion batteries. *Energy Storage Mater.* **32**, 281–289 (2020).

28. Z. Yang, Q. Shi, X. Yu, Y. Gu, Y. Liu, T. Zhang, Q. Huang, W. Feng, Y. Zhao, High-efficacy multi-sodium carboxylate self-sacrificed additives for high energy density sodium-ion batteries. *Energy Storage Mater.* **70**, 103511 (2024).

29. A. Jain, O. Shyue Ping, G. Hautier, W. Chen, W. D. Richards, S. Dacek, S. Cholia, D. Gunter, D. Skinner, G. Ceder, K. A. Persson, Commentary: The Materials Project: A materials genome approach to accelerating materials innovation. *APL Mater.* **1**, 011002 (2013).

30. Y. H. Xiao, L. J. Miara, Y. Wang, G. Ceder, Computational screening of cathode coatings for solid-state batteries. *Joule* **3**, 1252–1275 (2019).

31. M. A. Green, Intrinsic concentration, effective densities of states, and effective mass in silicon. *J. Appl. Phys.* **67**, 2944–2954 (1990).

32. P. Canepa, G. S. Gautam, D. Broberg, S.-H. Bo, G. Ceder, Role of point defects in spinel Mg chalcogenide conductors. *Chem. Mater.* **29**, 9657–9667 (2017).

33. P. Kastner, R. Hoppe, Die Kristallstruktur von $Na_6[ZnO_4]$. *Z. Anorg. Allg. Chem.* **409**, 69–76 (1974) [The Crystal Structure of Na6[ZnO4]].

34. H. Zentgraf, R. Hoppe, Na_5NiO_4, das erste oxoniccolat(III) mit inselstruktur. *Z. Anorg. Allg. Chem.* **462**, 61–70 (1980) [Na5NiO4, the First Oxonickelate(III) with Island Structure].

35. G. Brachtel, N. Bukovec, R. Hoppe, Das erste oxomanganat(III) mit inselstruktur: Zur Kenntnis von $Na_5[MnO_4]$. *Z. Anorg. Allg. Chem.* **515**, 101–113 (2004) [The First Oxomanganate(III) with Island Structure: On the Knowledge of Na5[MnO4]].

36. C. Jeannot, B. Malaman, R. Gérardin, B. Oulladiaf, Synthesis, crystal and magnetic structures of the sodium ferrate (IV) Na_4FeO_4 studied by neutron diffraction and mössbauer techniques. *J. Solid State Chem.* **165**, 266–277 (2002).

37. S. Koo, J. Lee, J. Lee, S. Yoon, D. Kim, Importance of metal-oxygen bond for stable oxygen-redox reaction in Li-excess layered oxides. *Energy Storage Mater.* **42**, 764–772 (2021).

38. R. A. Lewis, G. Wu, T. W. Hayton, Synthesis and characterization of an iron(IV) ketimide complex. *J. Am. Chem. Soc.* **132**, 12814–12816 (2010).

39. S. A. Cramer, D. M. Jenkins, Synthesis of aziridines from alkenes and aryl azides with a reusable macrocyclic tetracarbene iron catalyst. *J. Am. Chem. Soc.* **133**, 19342–19345 (2011).

40. T. Yamamoto, Assignment of pre-edge peaks in K-edge x-ray absorption spectra of 3d transition metal compounds: Electric dipole or quadrupole? *X-Ray Spectrom.* **37**, 572–584 (2008).

41. K. Jun, L. Kaufman, W. Jung, B. Park, C. Jo, T. Yoo, D. Lee, B. Lee, B. D. D. McCloskey, H. Kim, G. Ceder, Understanding the irreversible reaction pathway of the sacrificial cathode additive Li_6CoO_4. *Adv. Energy Mater.* **13**, 2301132 (2023).

42. N. Yabuuchi, H. Yoshida, S. Komaba, Crystal structures and electrode performance of alpha-$NaFeO_2$ for rechargeable sodium batteries. *Electrochemistry* **80**, 716–719 (2012).

43. V. Lacivita, Y. Wang, S.-H. Bo, G. Ceder, Ab initio investigation of the stability of electrolyte/electrode interfaces in all-solid-state Na batteries. *J. Mater. Chem. A* **7**, 8144–8155 (2019).

44. Y. Tian, T. Shi, W. D. Richards, J. Li, J. C. Kim, S.-H. Bo, G. Ceder, Compatibility issues between electrodes and electrolytes in solid-state batteries. *Energ. Environ. Sci.* **10**, 1150–1166 (2017).

45. S. P. Ong, W. D. Richards, A. Jain, G. Hautier, M. Kocher, S. Cholia, D. Gunter, V. L. Chevrier, K. A. Persson, G. Ceder, Python materials genomics (pymatgen): A robust, open-source python library for materials analysis. *Comput. Mater. Sci.* **68**, 314–319 (2013).

46. C. Zhan, Z. Yao, J. Lu, L. Ma, V. A. Maroni, L. Li, E. Lee, E. E. Alp, T. Wu, J. Wen, Y. Ren, C. Johnson, M. M. Thackeray, M. K. Y. Chan, C. Wolverton, K. Amine, Enabling the high capacity of lithium-rich anti-fluorite lithium-iron oxide by simultaneous anionic and cationic redox. *Nat. Energy* **2**, 963–971 (2017).

47. M. Wilke, F. Farges, P. E. Petit, G. E. Brown, F. Martin, Oxidation state and coordination of Fe in minerals: An Fe K-XANES spectroscopic study. *Am. Mineral.* **86**, 714–730 (2001).

48. Y. Li, Y. Gao, X. Wang, X. Shen, Q. Kong, R. Yu, G. Lu, Z. Wang, L. Chen, Iron migration and oxygen oxidation during sodium extraction from $NaFeO_2$. *Nano Energy* **47**, 519–526 (2018).

49. Y. Chen, Y. Zhu, Z. Sun, X. Kuai, J. Chen, B. Zhang, J. Yin, H. Luo, Y. Tang, G. Zeng, K. Zhang, L. Li, J. Xu, W. Yin, Y. Qiu, Y. Zou, Z. Ning, C. Ouyang, Q. Zhang, Y. Qiao, S.-G. Sun, Achieving high-capacity cathode presodiation agent via triggering anionic oxidation activity in sodium oxide. *Adv. Mater.* **36**, e2407720 (2024).

50. Y. Chen, M. Yang, Y. Zhu, J. Yin, L. Li, J. Xue, B. Zhang, H. Luo, K. Zhang, Z. Wu, Y. Tian, J. Xu, W. Yin, Q. Wang, N. Liu, Y. Sun, M. Yang, Y. Qiu, X. Sun, Y. Qiao, S.-G. Sun, Converting Li-rich layered oxide cathode into non-shrinking sacrificial prelithiation agent. *Adv. Mater.* **37**, e09827 (2025).

51. L. Qiu, H. Chen, X. Chen, S. Wang, Y. Zeng, Q. Zhang, D. Wu, J. Wang, J. Wang, F. Niu, In-situ catalytic decomposition of low-crystallinity $Li_2C_2O_4$ on Ni-rich cathodes via interfacial orbital hybridization. *Chem. Eng. J.* **525**, 170102 (2025).

52. C.-H. Jo, J. U. Choi, H. Yashiro, S.-T. Myung, Controllable charge capacity using a black additive for high-energy-density sodium-ion batteries. *J. Mater. Chem. A* **7**, 3903–3909 (2019).

53. C. Liu, H. Zhang, W. Zhou, X. Tian, T. Zhang, S. Niu, J. Li, M. Cao, Q. Wang, F. Lv, T. Peng, L. Tao, X. Rang, Z. Chen, X. Su, Air-stable Li_5FeO_4 additive enabled by carbon coating for energy-dense lithium-ion batteries. *Nat. Commun.* **16**, 7694 (2025).

54. L. Hu, J. Li, Y. Zhang, H. Zhang, M. Liao, Y. Han, Y. Huang, Z. Li, Enhancing the initial coulombic efficiency of sodium-ion batteries via highly active Na_2S as presodiation additive. *Small* **19**, e2304793 (2023).

55. P.-F. Wang, H.-R. Yao, X.-Y. Liu, Y.-X. Yin, J.-N. Zhang, Y. Wen, X. Yu, L. Gu, Y.-G. Guo, Na^+/vacancy disordering promises high-rate Na-ion batteries. *Sci. Adv.* **4**, eaar6018 (2018).

56. H. M. Rietveld, A profile refinement method for nuclear and magnetic structures. *J. Appl. Cryst.* **2**, 65–71 (1969).

57. B. H. Toby, R. B. Von Dreele, GSAS-II: The genesis of a modern open-source all purpose crystallography software package. *J. Appl. Cryst.* **46**, 544–549 (2013).

58. B. Ravel, M. Newville, ATHENA, ARTEMIS, HEPHAESTUS: Data analysis for X-ray absorption spectroscopy using IFEFFIT. *J. Synchrotron Rad.* **12**, 537–541 (2005).

59. X. Zhang, L. Guo, L. Gan, Y. Zhang, J. Wang, L. R. Johnson, P. G. Bruce, Z. Peng, LiO_2: Cryosynthesis and chemical/electrochemical reactivities. *J. Phys. Chem. Lett.* **9**, 327–327 (2018).

60. G. Kresse, J. Furthmuller, Efficiency of ab-initio total energy calculations for metals and semiconductors using a plane-wave basis set. *Comput. Mater. Sci.* **6**, 15–50 (1996).

61. S. Grimme, J. Antony, S. Ehrlich, H. Krieg, A consistent and accurate ab initio parametrization of density functional dispersion correction (DFT-D) for the 94 elements H-Pu. *J. Chem. Phys.* **132**, 154104 (2010).

62. A. Urban, D.-H. Seo, G. Ceder, Computational understanding of Li-ion batteries. *npj Comput. Mater.* **2**, 16002 (2016).

63. L. Hu, Y. Chen, Q. Zhang, S. Yang, H. Zhang, J. Peng, Y. Zhang, Z. Li, Y. Huang, Sodium acetate as residual-free presodiation additive for enhancing the energy density of sodium-ion batteries. *ACS Energy Lett.* **9**, 1148–1157 (2024).

64. J. Martinez De Ilarduya, L. Otaegui, J. M. López Del Amo, M. Armand, G. Singh, NaN3 addition, a strategy to overcome the problem of sodium deficiency in P2-$Na_{0.67}[Fe_{0.5}Mn_{0.5}]O_2$ cathode for sodium-ion battery. *J. Power Sources* **337**, 197–203 (2017).

65. M. Cao, L. Xu, Y. Guo, Y. Li, Q. Fang, Y. Liu, R. Bai, J. Zhu, Y. Gao, T. Cheng, J. Li, X. Wang, Y. Guo, Z. Wang, L. Chen, Air-stable $Na_{3.5}C_6O_6$ as a sodium compensation additive in cathode of Na-ion batteries. *Small* **20**, e2400498 (2024).

66. Z. Zhang, R. Zhang, R. Rajagopalan, Z. Tang, D. Sun, H. Wang, Y. Tang, A high-capacity self-sacrificial additive based on electroactive sodiated carbonyl groups for sodium-ion batteries. *Chem. Commun.* **58**, 8702–8705 (2022).
